# Trainability of affordance judgments in right and left hemisphere stroke patients

**DOI:** 10.1371/journal.pone.0299705

**Published:** 2024-05-03

**Authors:** Isabel Bauer, Lisa Finkel, Milena S. Gölz, Sarah E. M. Stoll, Joachim Liepert, Klaus Willmes, Jennifer Randerath

**Affiliations:** 1 Department of Psychology, University of Konstanz, Konstanz, Germany; 2 Lurija Institute for Rehabilitation Science and Health Research, Kliniken Schmieder, Allensbach, Germany; 3 Psychotherapy Training Center Bodensee (apb), Konstanz, Germany; 4 Faculty of Psychology, Department of Developmental and Educational Psychology, University of Vienna, Vienna, Austria; 5 Department of Neurology, University Hospital, RWTH Aachen University, Aachen, Germany; 6 Faculty of Psychology, Outpatient Unit for Research, Teaching and Practice, University of Vienna, Vienna, Austria; Universita di Bologna, ITALY

## Abstract

Whenever we are confronted with action opportunities in everyday life, e.g., when passing an opening, we rely on our ability to precisely estimate our own bodily capabilities in relation to the environmental conditions. So-called affordance judgments can be affected after brain damage. Previous studies with healthy adults showed that such judgments appeared to be trainable within one session. In the current study, we examined whether stroke patients with either right brain damage (*n* = 30) or left brain damage (*n* = 30) may similarly profit from training in an aperture task. Further, the role of neuropsychological deficits in trainability was investigated. In the administered task, stroke patients decided whether their hand would fit into a presented opening with varying horizontal width (Aperture Task). During one training session, patients were asked to try to fit their hand into the opening and received feedback on their decisions. We analyzed accuracy and the detection theory parameters perceptual sensitivity and judgment tendency. Both patients with right brain damage and patients with left brain damage showed improved performance during training as well as post training. High variability with differential profiles of trainability was revealed in these patients. Patients with impaired performance in a visuo-spatial or motor-cognitive task appeared to profit considerably from the target-driven action phase with feedback, but the performance increase in judgments did not last when the action was withdrawn. Future studies applying lesion analysis with a larger sample may shed further light on the dissociation in the trainability of affordance judgments observed in patients with versus without visuo-spatial or motor-cognitive deficits.

## Introduction

Properties of the environment afford certain actions. For instance, an object can be reachable or graspable, a chair can be suitable to sit on, and stairs can be climbable. However, action opportunities are also dependent on a person’s capabilities and physical conditions. Relative to both the object’s characteristics and one’s individual bodily limits, actions are performable and settings manipulable [1–4]. A small opening might only be suitable to put a child’s hand through, but not an adult’s. Thus, affordances can be regarded as action opportunities defined by the properties in the environment and the individual’s capabilities. Such judgments based on affordances play a key role in everyday life. In contrast to other studies focusing on affordances for object manipulation such as grasping or functional tool use (for a review see [5]), the current study focuses on actor-related abilities with the individual subject as major reference for deciding about action opportunities. These studies show that healthy young participants are able to adequately judge affordance action opportunities. This was shown for different tasks such as passing through an aperture [6–10], reaching [7,11–13], stepping across obstacles/ gaps [14,15] or navigating under barriers [6,16,17]. Nevertheless, affordance judgments (AJs) can involve mistakes. Incorrect decisions can quickly lead to severe consequences. The likelihood of dangerous accidents increases for example, when a person only slightly misjudges the opportunity to cross the street while a vehicle is approaching. Healthy young participants show relatively accurate, but not perfect affordance judgments. Whether they over- or underestimate the opportunity for a specific action depends on the type of task. For judging reachability, frequently a tendency to overestimate one’s capabilities has been demonstrated [e.g., 7,18], while the response behavior of participants judging fit for a given opening tends towards underestimation [e.g., 19,20].

There is evidence that stroke can have a negative impact on AJs [21,22]. Some studies suggest that assessed performance decreases go along with problems in everyday life, such as an increased risk of falls. For example, Takatori and colleagues [23] found that hemiplegic patients with multiple falls during hospitalization showed greater overestimation of maximum reaching distance of the non-affected side than patients with zero or only single falls. A study by Muroi and colleagues [24] linked a higher number of falls to more frequent collisions with the motor-affected side, when navigating through an aperture. In another study, stroke patients seemed to perform comparable to healthy controls in horizontal hand size estimation, but they performed worse than healthy controls when judging whether their hand would fit through apertures [21]. In a recent study by Randerath and colleagues [22], abnormal behavior in judgment tendency (i.e., rather liberal vs. rather conservative judgments) was found for left brain damage (LBD) patients compared to healthy controls when judging the reachability of an object. This atypical decision strategy, i.e., an instable judgment tendency appears to be associated with left lateralized ventro-dorsal regions [22]. In contrast, right brain damage (RBD) patients’ judgment tendency did not differ from healthy controls’. The potential underlying mechanisms of impaired AJs after stroke are several, as brain damage can have negative impact on diverse functions.

A broad framework helpful for drawing hypotheses about effects of brain damage and affected mechanisms is provided by Cisek [25] and Cisek and Kalaska [26]. The authors link arguments on the competition between action opportunities with neurophysiological data and propose a framework for affordance competition. This framework suggests a complex bilateral brain network with dynamic parallel action-related processes involving perception, cognition, and action. The model includes a dorsal posterior-anterior network focusing on spatial information processing, transforming visual information into representations of potential actions (action specification). The fronto-parietal cortex processes various information via different substreams (e.g., input from the basal ganglia) and deals with a competition between potential actions for further processing. Input from brain systems such as the ventral stream (sensitive to object identity information) further influences action selection. The authors propose that action selection and action specification processes occur simultaneously and that actions are not completely pre-planned when the chosen action is initiated [25,27,28]. The network of these affordance competition processes is likely disrupted by brain damage. Depending on the lesioned areas in the brain, this may affect subcomponents differentially.

As affordances reflect opportunities for action and need to be perceived and processed as such, neuropsychological deficits such as deficient action planning and deficient visuo-spatial perception may interfere with AJs. For example, stroke patients may suffer from limb apraxia, a disorder concerning difficulties in selecting and producing actions. Limb apraxia becomes apparent for example in pantomiming, imitating of gestures or tool use and multistep actions involving tools and objects [29–33]. Limb apraxia is linked to malfunctions in the fronto-temporo-parietal praxis network in the left hemisphere [34–38]. Based on the abovementioned affordance competition hypothesis [25], Rounis and Humphreys [39] state that some aspects regarding apraxia may result from a deviation in sensitivity to competing affordances or poor cognitive control over the competition between multiple present affordances.

Further, intact visuo-spatial capabilities play a role in affordance tasks. Most frequently, lesions in the right hemisphere are associated with deficits such as neglect, which becomes apparent in visuo-spatial tasks [40–42]. Lesions associated with neglect are typically located in the fronto-parietal network that has been suggested to be important for the processing of affordance action opportunities by Cisek [25]. Indeed, correlations have been found between these neuropsychological deficiencies and lowered performance in AJs as well as, on a neuroanatomical level, between deficiencies in AJs and lesions in the fronto-parietal network [21,22].

The described difficulties stroke patients may encounter in AJ tasks suggest a need for trainings. But is AJ performance in stroke patients trainable? Thus far, very little is known about the trainability of AJs in stroke patients. In healthy participants, practice and experience may improve judgments in the AJ tasks [7,8,43–46]. For the trainability of AJs in an Aperture Task, Finkel and colleagues [8] revealed significant increases in accuracy after a training session as well as at follow-up. This could be shown for both young and elderly adults. However, the groups showed qualitative differences in their improved judgments. While the young group improved in perceptual sensitivity, the elderly adjusted their judgment tendency towards less conservative judgments. These study results show that healthy people are able to benefit from training. As trainable aspects appear to depend on features of the sample, it is less clear to what extent individuals with brain damage (e.g., due to stroke) can profit from training sessions to improve their AJs in for example an Aperture Task. In general, stroke patients with impairment in motor-cognitive tasks (e.g., due to apraxia) seem to be able to profit from training activities of daily living and from gesture training (for a review see [47]). In the past decade, the relationship between limb apraxia and affordances has been discussed particularly with a focus on how objects can provide affordances for appropriate actions [48]. For example, tools with less degrees of freedom regarding the opportunities for their usage seem to benefit apraxic patients’ performance [49]. Particularly for these patients, it seems that settings with clear information about defined hand-object interactions may reduce the number and load of competing action opportunities and thereby ease performance. Especially the immediate anticipation of a concrete action combined with online feedback might help specify this action (as it is clearly defined) and facilitate cognitive control. By reducing affordance competition there might be more capacities for sustainably learning a specific action.

Defined actions may also be beneficial for patients with impairment in visuo-spatial tasks, i.e., due to neglect. In their review, Harvey and Rossit [50] conclude that whereas patients with neglect show impairments in delayed reaching and grasping, target directed on-line actions do not seem to be affected. For example, in a study by Humphreys and Riddoch [51], patients were instructed to find an object cued either by its name (“find the cup”) or by its related action (“find the object to drink from”). Cuing the object in an action-related manner appears to lead to improved performance, e.g., less apparent neglect [52]. Particularly for these patients, evoking the anticipation of an action, e.g. by letting people try to stick their hand into a given opening after a judgment on the possibility of this action, may bind the attention towards experiencing a fit or non-fit.

In summary, when it comes to rather actor-related judgments based on affordances¸ patients with impairment in motor-cognitive tasks (e.g., due to limb apraxia) or impairment in visuo-spatial tasks (e.g., due to neglect) appear to perform worse compared to healthy controls [21,22]. But when being active in hand-object interactions such as tool use, object-related affordances have been demonstrated to provide helpful context for patients with motor cognitive deficits (apraxia) [49] as well as patients with visuo-spatial deficits (neglect) [52]. Therefore, we would expect that when patients with such deficits are allowed to act upon a defined environment (such as the here applied Aperture Task), judgment performance may be improved and perhaps even be trainable.

For this purpose, we applied an affordance judgment task (adapted from [53]) that has further been adapted by Randerath and Frey [7] taking challenges such as motor impairments or aphasia into account, e.g., by simple instructions and the possibility to execute the task with only one hand. In this Aperture Task, participants are asked to decide whether their hand would fit into a presented opening with varying horizontal size. In the present study, we examine potential training effects in stroke patients. We applied a Signal Detection Theory (SDT) approach [54–56] to analyze our data (S1 Dataset). In contrast to general accuracy measures, this approach allows to separate perceptual sensitivity and potential judgment tendency (i.e., response bias), leading to a more specific description of performance [57].

We hypothesize that both RBD and LBD stroke patients improve in accuracy, perceptual sensitivity and judgment tendency in the affordance Aperture Task by means of a short training session both during training and after training (Hypothesis 1). More specifically, we assume that both patients with and without motor-cognitive (impairment in imitation of gestures) or visuo-spatial deficits (impairment in star cancellation) improve significantly during training (Hypothesis 2). As patients with neglect only seem to profit from action-related information, it might be that the subgroup of RBD patients with visuo-spatial deficits (impairment in star cancellation) shows a significant decline in accuracy, perceptual sensitivity and judgment tendency post training when action-related feedback is withdrawn (Hypothesis 3). Since it has been demonstrated that patients with motor-cognitive deficits (apraxia) can be trained in activities of daily living including the selection of tools and objects, we assume that all LBD patients can profit from the training even when action-related feedback is removed (Hypothesis 4).

## Methods

The project was conducted in accordance with the Declaration of Helsinki and was approved by the Ethics Committee of the University of Konstanz (#02/2016). The study was performed in cooperation with the Lurija Institute of the Kliniken Schmieder in Allensbach, Germany. Before taking voluntarily part in the study, the participants gave informed written consent.

The main task (affordance judgment task: Aperture Task) of the study was administered in two sessions, whenever possible without interfering with therapy plans within two consecutive weekdays and at the same time of the day, mostly in the afternoon. Both sessions lasted approximately 45–60 minutes each. The first session consisted of familiarization with the task and an initial diagnostic assessment. In the second session the diagnostic assessment was repeated before feedback as well as post feedback.

## Participants

Participants included in the present study were tested during their neurological rehabilitation stay at Kliniken Schmieder in Allensbach between October 11, 2016 and June 28, 2022. In the beginning of data collection we calculated the minimum number of required participants using G-power [58]. Power analysis was based on descriptive data of the first participants (RBD: *n* = 7, LBD: *n* = 11,) and resulted in a sample size suggestion of *n* = 20 RBD patients (within-subject design, pre vs. post, power: 0.96, effect size: d = 0.78) and *n* = 30 LBD patients (within-subject design, pre vs. post, power: 0.96, effect size: d = 0.63).

In sum, 60 patients with unilateral stroke participated. Half of the individuals suffered from right brain damage (RBD) and half suffered from left brain damage (LBD). The side of brain damage was determined from each patient’s medical reports. The lesion location has been verified in the MRT or CT brain scans for all patients except for five patients, for whom no brain scans were available. All participants reported no history of other neurologic or psychiatric disorders, were right-handed according to the lateralization quotient [59] and had normal or corrected-to-normal vision. RBD patients (*n* = 30, 12 female) had a mean age of *M* = 60.03 years (*SD* = 12.10) and a mean of 90 days since stroke onset (range: 28–392). LBD patients (*n* = 30, 13 female) had a mean age of *M* = 62.13 years (*SD* = 13.06) and a mean of 67 days since stroke onset (range: 20–175). In order to evaluate training effects in patients with impairment in a visuo-spatial task, we split the RBD group into one subgroup with impairment in a visuo-spatial task (*n* = 15; age: *M* = 61.00 years, *SD* = 9.17; 5 female) and one subgroup without impairment in a visuo-spatial task (*n* = 15; age: *M* = 59.07 years, *SD* = 14.74; 7 female), based on their performance in the subtest star cancellation of the Behavioral Inattention Test [BIT; 60]. RBD patients with a score below the cut-off of 51 out of 54 target stimuli were assigned to the subgroup with impairment in star cancellation. In order to evaluate training effects in patients with impairment in a motor-cognitive task, we split the LBD group into one subgroup with impairment in a motor-cognitive task (*n* = 15, age: *M* = 63.93 years, *SD* = 13.39; 6 female) and one without impairment in a motor-cognitive task (*n* = 15, age: *M* = 60.33 years, *SD* = 12.93; 7 female), based on their performance in the subtest imitation of meaningless hand gestures of the Diagnostic Instrument of Limb Apraxia–short version [DILA-S; 61]. Patients with a hand imitation score below the cut-off of 16 (21–50 years old) or the cut-off of 15 (51–80 years old) were assigned to the subgroup with impairment in hand gesture imitation. See S1 Table for a more detailed description of the groups and subgroups. All participants used their unaffected ipsilesional hand to indicate their judgment responses via button press, as patients with hemiparesis were included.

In order to provide a performance reference with baseline measures for the current study, we additionally assessed 15 healthy controls, 9 with their right hand as button-pressing hand and 6 with their left hand as button-pressing hand, which is the hand that has been judged to fit or not to fit into a given opening (equivalent to the ipsilesional hand in patients). In this healthy subjects group, performance appeared not to be influenced by whether the relevant hand was the left or the right hand (see S6 Table, between subjects comparisons), which goes along with previous study results [8]. We therefore merged the control participants into one group when reporting performance in healthy control participants. For additional statistical comparisons between the healthy control group and stroke patients reported in the supporting information, we compared judgments for the button-pressing hand between controls and a subgroup of patients matched with respect to hand used/ judged for, age and gender (see S3 Text). The healthy control group consisted of 4 female and 11 male participants and had a mean age of *M* = 55.27 (*SD* = 14.92).

### Material

**Fig 1:**
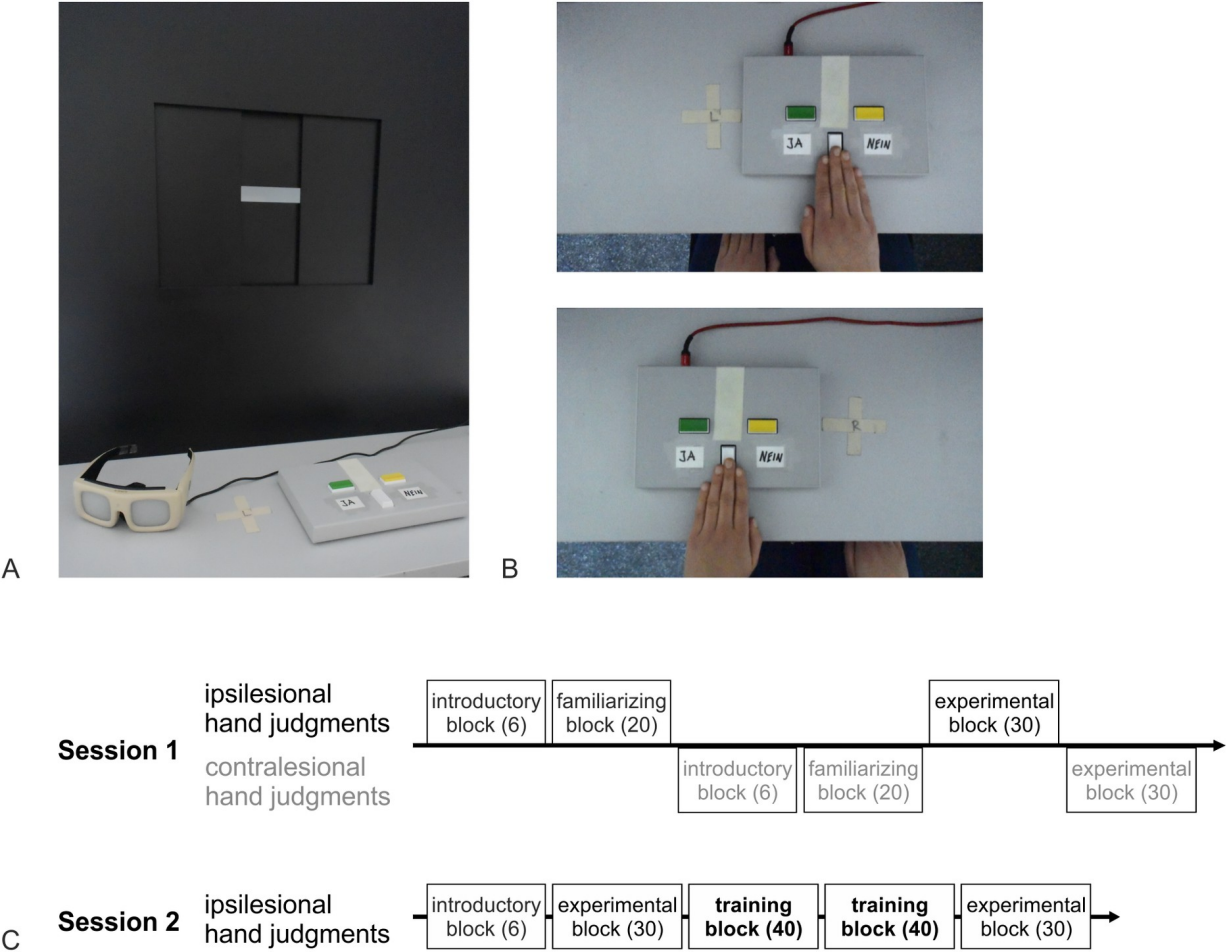
Experimental setting and procedure of the applied Aperture Task. (A) Experimental setting included Aperture apparatus, button-pad, goggles (here: opaque). (B) The exemplary image above shows the setting for RBD patients (judging whether their right hand would fit into the aperture). The exemplary image below shows the setting for LBD patients (judging whether their left hand would fit into the aperture). (C) Overview of the procedure in Session 1 and Session 2. The numbers in brackets represent the number of trials within each block. Note that two additional extreme demonstration trials preceded each experimental block. Please note that contralesional hand judgments (shown grayed out) are not of specific interest for the present study and are not included in the data analysis. *Note*. The used material is identical to the material presented in our previous study [8]. The here displayed illustrations were photographed by Isabel Bauer.

Material and measurement procedure were adapted from previous studies [7,21,44]. The Aperture Apparatus is custom-made and was built on a height adjustable table in order to present the variable rectangular openings at the participant’s eye level. Regulation of the rectangle’s width for each trial was carried out by a computer-controlled motor and followed a standardized trial protocol. Superlab 5 Software (provided by Cedrus) was used for experimental data coding. For both the size perception and the AJ task (Aperture Task) the participants’ individual hands served as stimuli. Participants gave their response on a two-button response pad (Cedrus, RB540) with a green button marked “Yes” and a yellow button marked “No”. Further, participants were equipped with Plato goggles (Translucent Technologies Inc.), allowing to switch between opaque and transparent in order to control vision. This enabled to prevent visual feedback between trials and during the measurement of the actual hand width. See Fig 1A for illustration of the experimental material and setting.

### Tasks and procedure

Measurements. Participants’ hands were measured in both sessions. The first session started with the measurement of the ipsilesional hand’s maximum width (horizontal opening; measured around proximal phalanges and metacarpal bones) and height (vertical opening; measured from palm to dorsum of the hand). For this purpose, the opening was tightly closed around the flat hand’s widest part with all five fingers closely put together. While haptic feedback was available, visual feedback was avoided by use of shut Plato goggles worn by the participant. The measurement of the contralesional hand took place right before the onset of the corresponding block. In most patients, the procedure for measuring the contralesional hand was the same as for the ipsilesional hand. In patients with a severely paretic contralesional arm, a digital caliper (hardware store Toom) was applied. The measurements were checked twice for each hand to avoid measurement errors.

Aperture task. In this affordance perception task participants had to estimate and state whether they would be able to put their hand through a presented aperture. Participants were instructed to respond as precisely as possible. The vertical opening remained at the actual vertical hand size, while the opening’s width either differed in fixed randomized increments from the individual’s actual hand width or reflected the exact hand size (set of increments: -16, -8, -4, -2, ±0, +2, +4, +8, +16 mm). Thus, the 0-trial represented the minimum opening size the hand fitted through. Per set of increments one filler trial (-20, -30 or -40 mm) was included for which the correct answer would be “no” to achieve a balanced number of yes-trials (fit) and no-trials (non-fit). As the filler trials were more extreme trials with a high likelihood to be judged correctly, they were excluded from further analysis.

#### First session

To introduce participants to the general task procedure including yes/ no button presses and to check task comprehension, 6 trials with extremely small or large openings were presented. Afterwards, an introductory set of 20 trials was presented, because earlier studies indicate a familiarization phase during which a rather stable judgment tendency is established [7]. The experimental blocks consisted of 27 trials of interest (3x9 openings), completed with three filler trials, resulting in 30 trials per experimental block. Before each experimental block, 2 extreme demonstration trials for which the correct response was obvious were administered to ensure correct understanding of the task (not included in analysis). The frequency of the presented increments was the same within each block as well as between blocks. The sequence of blocks is depicted in Fig 1C.

Participants were reminded of which hand the openings should be judged for before each block. When the judgment had to be given for the ipsilesional hand (e.g., button-pressing hand; right hand for RBD patients, left hand for LBD patients), the contralesional hand was lying on the participants’ thighs. The marked spot indicating the hand’s required position was set slightly aside from the aperture’s midline to prevent direct alignment relative to the opening. The participants gave their responses pressing a “yes” or “no” button on the response pad. Please note that we only analyzed judgments for the ipsilesional hand. Data for the contralesional hand have been gathered in Session 1. This data was not collected in the training session and is not of specific interest for this study. Therefore, this data was not analyzed.

#### Second session (training)

The second session involved a short feedback training in order to examine whether the participants’ AJs would improve with experience. For each trial, participants were asked to actually try and fit their ipsilesional (unaffected) hand through the openings right after their judgment. In case of an actual fit, participants received haptic and acoustic feedback automatically by reaching and touching a back-board with a sensor. The back-board was adjusted to the participants’ individual hand-length and triggered a validation sound when it was pressed. Feedback training consisted of two consecutive blocks with 40 trials each. Before and after the two consecutive feedback blocks, 30 experimental trials were performed respectively (Fig 1C). Similar to the first session, each experimental block was introduced by two additional extreme demonstration trials. All of the judgments in the second session were made for the ipsilesional hand.

Additionally, two control tasks to measure size estimation performance and reaction times were administered. Please see S1 Text for size estimation task methods and results and S2 Text for reaction time task methods and results. Simple reaction times were assessed to obtain a measure of alertness.

### Data analysis

Behavioral data were analyzed with SPSS 28 (IBM) and JASP [version 0.16.3.0; 62]. Missing trials, e.g., no recordable response by the participants, were rare (< 0.3% in total).

Performance variables. In addition to accuracy (percent correct) we analyzed the two independent signal detection theory variables perceptual sensitivity and judgment tendency [54,56]. The perceptual sensitivity measure d’ represents the ability of the participants to discriminate a fit from a non-fit. Higher scores stand for better performance. The judgment tendency or criterion c indicates whether the participants decide rather liberal (negative values of c; i.e., opt for a fit more often than the ideal observer) or conservative (positive values of c; i.e., opt for a non-fit more often than the ideal observer). The calculation of both perceptual sensitivity d’ and judgment tendency c takes Hit and False-Alarm rates into account (d’ = *z*(Hit rate)–*z*(False-Alarm rate); c = -0.5 × [*z*(Hit rate) + (*z*(False-Alarm rate)]. For judgment tendency calculations, we used absolute values of judgment tendency (c) to analyze the deviation from an ideal criterion (which equals 0). More accurate judgments are represented by higher values for accuracy and perceptual sensitivity, but by lower absolute values for judgment tendency.

Screening of normal probability plots and descriptives on skewness and kurtosis as well as the results of Shapiro-Wilk tests indicated that part of the residuals of our dependent variables (accuracy, perceptual sensitivity, judgment tendency) were not normally distributed in different subgroups (see S2 Table for Shapiro-Wilk test results). For this reason, behavioral data were analyzed by use of non-parametric procedures. Exact instead of asymptotic p-values were reported two-tailed when not further specified.

Based on the reviewer comments, we provide also parametric analyses. Similar results were obtained (see S7–S11 Tables).

#### Within-subject analyses

According to our hypotheses, we ran Friedman tests both in the RBD and LBD group to investigate a main effect of timepoint (pre training, training, post training) per variable (perceptual sensitivity, accuracy, judgment tendency). Based on significant results, post-hoc Wilcoxon signed ranks tests were applied to further specify the effect of training during the training session with feedback as well as after training, when feedback was no more available. The same procedures were applied for the subgroup analyses of patients with impairment in star cancellation or imitation of gestures to test for training effects (see previous section “Participants”).

Using the Bonferroni procedure, we adjusted p-values (*p*_*adj*_) to correct for family-wise error rate per variable and (sub)group. Corresponding z-values of post-hoc tests were used to calculate the effect size *r* as proposed by Cohen [63] by dividing *z* by the square root of *n*. Please note that *n* corresponds to the number of observations (total group analyses: *n* = 60, subgroup analyses: *n* = 30) [64].

#### Between-subject analyses

For the subgroup analyses, we ran Mann-Whitney tests to investigate baseline differences between subgroups with and without neuropsychological deficits. Further, to determine a value for change across timepoints, we calculated difference scores (training—pre training; post training—training; post training—pre training) in each subgroup. We then ran Mann-Whitney tests to investigate interactions between change across timepoints and the respective subgroups (with vs. without impairment in star cancellation, with vs. without impairment in imitation of meaningless gestures) in order to evaluate differences on the one hand in improvement with training and on the other hand in decline when feedback was withdrawn. Effect size *r* was calculated for between subjects analysis in the same way as for post-hoc within-subject analyses.

#### Supplemental report of Bayes factors

As Bayesian statistics enable to calculate evidence for the null hypothesis [65], we report Bayes factors additionally to standard Wilcoxon signed ranks or Mann-Whitney tests. Where possible and according to our hypotheses, we calculated Bayes factors with a directed alternative hypothesis. This was the case for calculations regarding pure repetition effects (alternative hypothesis for accuracy and perceptual sensitivity: baseline < pre training, *BF*_*0-*_; alternative hypothesis for judgment tendency: baseline > pre training, *BF*_*0+*_) as well as when testing for the preservation of the training gain from active training to post training in the subgroup without impairment in star cancellation and in the subgroups with and without impairment of gesture imitation (alternative hypothesis for accuracy and perceptual sensitivity: training > post training, *BF*_*0+*_; alternative hypothesis for judgment tendency: training < post training, *BF*_*0-*_) and a training loss in the subgroup with impairment in star cancellation (alternative hypothesis for accuracy and perceptual sensitivity: training > post training, *BF*_*+0*_; alternative hypothesis for judgment tendency: training < post training, *BF*_*-0*_). Also, Bayes factor calculations for training gain (pre training to training; pre training to post training) were applied with a directed alternative hypothesis (alternative hypothesis for accuracy and perceptual sensitivity: Measure 1 < Measure 2, *BF*_*-0*_; alternative hypothesis for judgment tendency: Measure 1 > Measure 2, *BF*_*+0*_). Bayes factor calculation was also used when testing for age differences, differences in Barthel Index or baseline differences between groups. Here, no specific alternative hypothesis was assumed (alternative hypothesis: Measure 1 ≠ Measure 2, *BF*_*01*_). For all calculations of Bayes Factors, we used the default prior scale value in JASP (Cauchy scale: .707).

As an example, for the interpretation of Bayes factors [e.g., 66,67], a Bayes factor of *BF*_*10*_ = 15 is in favor of the alternative hypothesis with the data being 15 times more likely under the alternative hypothesis than under the null hypothesis. A Bayes factor of *BF*_*01*_ = 15, however, would indicate that the data is 15 times more likely under the null hypothesis. Bayes factors *BF*_*01*_ below 1 reflect rather support for H_1_ instead of H_0_. For example, *BF*_*01*_ = 0.04 is equivalent to *BF*_*10*_ = 1/ 0.04 = 25, meaning that the data are 25 times more likely under the assumption of H_1_ instead of H_0._ Accordingly, Bayes factors *BF*_*10*_ below 1 reflect support for H_0_ instead of H_1_.

## Results

First, we analyzed control task results and tested for replications of previous results. For control task results (size estimation task, reaction time task) and a comparison to a healthy control sample, see the supporting information (S1 Text, S2 Text and S3 Text). We then tested for pure effects of task repetition and analyzed potential group differences to reveal sample characteristics.

### Mere effects of task repetition

In order to analyze potential changes in judgment behavior merely due to the repetition of the experimental Aperture Task, we compared the baseline assessment in the first session and the pre training assessment in the second session by use of Wilcoxon signed ranks tests. Test results and Bayes factors are depicted in S3 Table. Participants did not improve in performance due to mere repetition. The results observed in the LBD group showed rather a slight decline in accuracy from baseline assessment to pre training assessment (baseline: *M*_*dn*_ = 77.78%, pre training: *M*_*dn*_ = 72.22%) as well as a decline in judgment tendency. Similarly, there was no improvement due to mere repetition neither in the RBD subgroups with or without impairment in star cancellation nor in the LBD subgroups with or without impairment in gesture imitation. In the LBD subgroup with impairment in gesture imitation, a decline in accuracy was apparent (baseline: *M*_*dn*_ = 81.48%, pre training: *M*_*dn*_ = 74.07%) as well as a decline in judgment tendency. Due to the significant decline in accuracy and judgment tendency performance in LBD patients, we merged the baseline assessment from the first session and the pre training assessment from the second session to one overall pre training measure for all patients, in order to avoid overestimating the expected training gain. The resulting Bayes factors (*BF*_*0-*_ > 5 for accuracy and perceptual sensitivity; *BF*_*0+*_ > 6 for judgment tendency) indicate that the probability of the data is more likely under the assumption of H_0_ (no improvement by mere repetition) than under the assumption of H_1_ (improvement by mere repetition). This support can be interpreted as strong in the entire group and as substantial in the subgroups [see 67]. S1 Fig provides boxplots showing the time course of performance in the familiarizing and the two experimental pre training blocks.

### Group characteristics and comparison of pre training performance

RBD and LBD patient groups did not differ significantly in age, time since stroke onset nor in their Barthel Index which was assessed by the clinic personnel. Similarly, there was no difference in age nor Barthel Index between the subgroups with and without impairment in star cancellation or hand gesture imitation. The subgroups with and without impairment in hand gesture imitation did not differ in time since stroke onset neither. The subgroups with and without impairment in star cancellation differed significantly in time since stroke onset, with patients with impairment in star cancellation showing longer time since stroke onset (see S4 Table for Mann-Whitney test results and Bayes factors for the group comparisons).

Comparisons of pre training performance between patient groups revealed that RBD patients showed worse accuracy and perceptual sensitivity performance than LBD patients pre training, but no significant difference in judgment tendency (see S4 Table for statistics and Table 1 for descriptive data).

**Table 1:** Descriptive data for RBD and LBD groups as well as their subgroups for the three timepoints.

		Timepoint		
		pre training	training	post training
Variable	Group	*M*_*dn*_ *[IQR]*	*M*_*dn*_ *[IQR]*	*M*_*dn*_ *[IQR]*
accuracy (in %)	RBD	68.52 [55.09, 74.54]	83.33 [78.82, 88.89]	79.63 [66.67, 85.19]
	not impaired star cancellation	62.96 [53.70, 72.38]	87.30 [81.71, 90.28]	84.62 [81.48, 88.89]
	impaired star cancellation	70.37 [55.56, 75.93]	83.10 [77.78, 84.72]	74.07 [62.96, 77.78]
	LBD	74.07 [68.52, 83.33]	87.50 [83.26, 89.24]	81.48 [77.78, 88.89]
	not impaired gesture imitation	73.43 [68.52, 83.33]	88.89 [83.33, 91.67]	85.19 [81.48, 88.89]
	impaired gesture imitation	79.63 [70.37, 83.33]	87.50 [81.94, 88.89]	81.48 [77.78, 85.19]
	HC	77.78 [70.37, 81.48]	88.89 [86.11, 90.28]	88.89 [81.48, 88.89]
perceptual sensitivity (d’)	RBD	1.14 [0.74, 1.59]	2.11 [1.85, 2.57]	1.82 [1.30, 2.25]
	not impaired star cancellation	1.12 [0.59, 1.71]	2.32 [1.97, 2.68]	2.08 [1.80, 2.43]
	impaired star cancellation	1.15 [0.85, 1.57]	2.02 [1.68, 2.27]	1.33 [1.14, 1.85]
	LBD	1.77 [1.38, 2.23]	2.48 [2.11, 2.69]	2.08 [1.80, 2.54]
	not impaired gesture imitation	1.65 [1.32, 2.26]	2.49 [1.97, 2.90]	2.32 [2.00, 2.56]
	impaired gesture imitation	1.88 [1.40, 2.22]	2.47 [2.16, 2.56]	1.92 [1.66, 2.15]
	HC	1.83 [1.68, 2.00]	2.52 [2.28, 2.78]	2.53 [1.92, 2.59]
judgment tendency (c)	RBD	0.40 [-0.35, 1.38] 0.95 [0.36, 1.38]	0.16 [-0.01, 0.30] 0.27 [0.12, 0.44]	0.21 [-0.33, 0.78] 0.43 [0.28, 0.94]
	not impaired star cancellation	1.10 [-0.10, 1.46] 1.10 [0.80, 1.46]	0.22 [-0.12, 0.36] 0.34 [0.12, 0.58]	0.28 [-0.26, 0.45] 0.40 [0.26, 0.84]
	impaired star cancellation	0.09 [-0.39, 1.26] 0.47 [0.32, 1.26]	0.14 [0.03, 0.29] 0.22 [0.11, 0.38]	-0.08 [-0.34, 1.10] 0.60 [0.32, 1.10]
	LBD	0.22 [-0.77, 0.64] 0.66 [0.39, 0.95]	-0.07 [-0.30, 0.22] 0.23 [0.14, 0.38]	-0.06 [-0.42, 0.42] 0.40 [0.23, 0.62]
	not impaired gesture imitation	0.08 [-0.75, 0.83] 0.75 [0.40, 1.22]	-0.05 [-0.23, 0.22] 0.22 [0.14, 0.34]	-0.09 [-0.46, 0.31] 0.34 [0.14, 0.72]
	impaired gesture imitation	0.24 [-0.84, 0.63] 0.63 [0.24, 0.88]	-0.08 [-0.37, 0.22] 0.28 [0.11, 0.38]	-0.04 [-0.40, 0.48] 0.40 [0.31, 0.52]
	HC	0.66 [-0.25, 0.91] 0.83 [0.25, 0.94]	0.05 [-0.33, 0.24] 0.24 [0.06, 0.38]	0.14 [-0.52, 0.45] 0.46 [0.28, 0.52]

*Note*. The baseline assessment in the first session and the pre training assessment in the second session were merged to one pre training measure. *Please note*. The second line for each group of the variable judgment tendency indicates the *M*_*dn*_ and *IQR* for the absolute judgment tendency values (absolute difference to an ideal criterion of 0).

### Effects of the intervention

Next, we analyzed potential changes due to the intervention according to our hypotheses. The main variable of interest was perceptual sensitivity (ability to discriminate a fit from a non-fit), as a previous study demonstrated that perceptual sensitivity can be diminished in this task in stroke patients, particularly in those with impairment in a motor-cognitive or visuo-spatial task [21].

**Fig 2:**
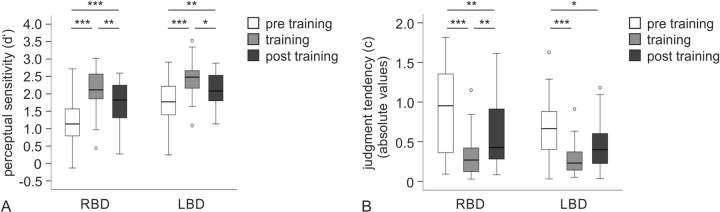
Boxplots for signal detection measures. (A) Boxplots for perceptual sensitivity (d’) per group. Higher values reflect better perceptual sensitivity. (B) Boxplots for judgment tendency (c). Absolute values closer to zero reflect a more ideal judgment tendency. *Note*. * *p* < .05, ** *p* < .01, *** *p* < .001 (after Bonferroni adjustment).

For perceptual sensitivity, both in the RBD and in the LBD group, patients achieved higher scores during and post training compared to pre training assessment. The Friedman test revealed a main effect of timepoint (pre training, training, post training) for both patient groups (RBD: χ^2^(2) = 29.27, *p* < .001; LBD: χ^2^(2) = 20.87, *p* < .001). Post-hoc Wilcoxon signed ranks tests showed significant differences between all three timepoints for the two patient groups respectively (see Fig 2A and Table 2 for statistics). Both RBD and LBD patient groups performed better during the training session compared to the pre training and still showed significantly better perceptual sensitivity post training when feedback was withdrawn as compared to pre training. However, both groups showed a significant decline in the performance gain in the post training assessment compared to when actively receiving the training.

**Table 2:** Post-hoc analyses comparing pre training performance with training and post training performance per group for accuracy (acc), perceptual sensitivity (d’) and judgment tendency (c).

		pre training vs. training					pre training vs. post training					training vs. post training				
Group	Variable	*z*	*p*_*ex*_	*p*_*adj*_	*r*	*BF*	*z*	*p*_*ex*_	*p*_*adj*_	*r*	*BF*	*z*	*p*_*ex*_	*p*_*adj*_	*r*	*BF*
RBD	acc	4.43	< .001	< .001	0.57	1881.14 (*BF*_*-0*_)	3.98	< .001	< .001	0.51	683.58 (*BF*_*-0*_)	2.91	.003	.008	0.38	0.02 (*BF*_*0+*_)
	d’	4.45	< .001	< .001	0.57	878.89 (*BF*_*-0*_)	4.00	< .001	< .001	0.52	358.25 (*BF*_*-0*_)	2.89	.003	.009	0.37	0.01 (*BF*_*0+*_)
	c	4.43	< .001	< .001	0.57	1307.34 (*BF*_*+0*_)	3.08	.001	.004	0.40	39.99 (*BF*_*+0*_)	3.28	< .001	.002	0.42	0.01 (*BF*_*0-*_)
LBD	acc	4.25	< .001	< .001	0.55	806.91 (*BF*_*-0*_)	3.63	< .001	< .001	0.47	422.54 (*BF*_*-0*_)	2.72	.005	.016	0.35	0.02 (*BF*_*0+*_)
	d’	3.88	< .001	< .001	0.50	472.36 (*BF*_*-0*_)	2.97	.002	.007	0.38	64.18 (*BF*_*-0*_)	2.52	.011	.032	0.33	0.07 (*BF*_*0+*_)
	c	4.39	< .001	< .001	0.57	2810.42 (*BF*_*+0*_)	2.73	.005	.016	0.35	33.18 (*BF*_*+0*_)	2.21	.026	.079	0.29	0.17 (*BF*_*0-*_)

*Note*. *p*_*adj*_ = Bonferroni adjusted p-values.

*Please note*. Bayes factors *BF*_*+0*_ (acc, d’) and *BF*_*-0*_ (c) reflect support for the alternative hypothesis (better performance during training). *BF*_*0+*_ and *BF*_*0-*_ reflect support for the null hypothesis (no decline in performance after training). Bayes factors *BF*_*0+*_ and *BF*_*0-*_ < 1 provide support for the alternative hypothesis.

For accuracy, results were consistent with perceptual sensitivity results (Friedman test: RBD: χ^2^(2) = 27.47, *p* < .001; LBD: χ^2^(2) = 25.29, *p* < .001; see Table 2 for post-hoc test results). As for perceptual sensitivity and accuracy, Friedman tests for judgment tendency revealed a significant main effect of timepoint for both the RBD (χ^2^(2) = 28.07, *p* < .001) and the LBD group (χ^2^(2) = 17.27, *p* < .001). Results are depicted in Fig 2B. Especially during but also after training, patients showed a more ideal judgment tendency closer to zero, compared to the pre training assessment.

Training gain (pre training to post training) did not correlate significantly with time since stroke onset in the patient groups. See S5 Table for statistics.

### Subgroup analysis

Subsequently, we examined the training effect in subgroups of RBD patients with versus without impairment in star cancellation and of LBD patients with versus without impairment in gesture imitation. The descriptive data is depicted in Table 1.

### RBD patients with impairment in star cancellation

Friedman tests revealed a significant difference between timepoints for both subgroups for perceptual sensitivity (without impairment in star cancellation: χ^2^(2) = 20.13, *p* < .001; with impairment in star cancellation: χ^2^(2) = 10.80, *p* = .003), accuracy (without impairment in star cancellation: χ^2^(2) = 19.73, *p* < .001; with impairment in star cancellation: χ^2^(2) = 11.20, *p* = .003) and judgment tendency (without impairment in star cancellation: χ^2^(2) = 15.60, *p* < .001; with impairment in star cancellation: χ^2^(2) = 14.93, *p* < .001). For post-hoc test statistics see Table 3. For all three variables, the subgroup without and the subgroup with impairment in star cancellation showed improved performance during training compared to the pre training assessment. Bayes factors are in line with these results and provide decisive evidence for an improvement in patients without impairment in star cancellation and very strong to decisive evidence in patients with impairment in star cancellation [see 67].

**Table 3:** Post-hoc analyses comparing pre training performance with training and post training performance per subgroup for RBD (a.) and LBD (b.) patients.

		pre training vs. training					pre training vs. post training					training vs. post training				
Group	Var.	*z*	*p*_*ex*_	*p*_*adj*_	*r*	*BF*	*z*	*p*_*ex*_	*p*_*adj*_	*r*	*BF*	*z*	*p*_*ex*_	*p*_*adj*_	*r*	*BF*
a. RBD																
not impaired star canellation	acc	3.35	< .001	< .001	0.61	364.27 (*BF*_*-0*_)	3.41	< .001	< .001	0.62	330.97 (*BF*_*-0*_)	1.73	.085	.256	0.32	0.31 (*BF*_*0+*_)
	d’	3.41	< .001	< .001	0.62	2184.32 (*BF*_*-0*_)	3.35	< .001	< .001	0.61	361.71 (*BF*_*-0*_)	1.93	.055	.166	0.35	0.20 (*BF*_*0+*_)
	c	3.24	< .001	.001	0.59	150.08 (*BF*_*+0*_)	3.18	< .001	.001	0.58	351.41 (*BF*_*+0*_)	1.48	.151	.454	0.27	0.47 (*BF*_*0-*_)
impaired star cancellation	acc	2.78	.003	.010	0.51	64:97 (*BF*_*-0*_)	1.82	.070	.211	0.33	2.83 (*BF*_*-0*_)	2.36	.016	.047	0.43	13.09 (*BF*_*+0*_)
	d’	2.78	.003	.010	0.51	66.96 (*BF*_*-0*_)	2.05	.041	.124	0.37	6.18 (*BF*_*-0*_)	2.39	.015	.045	0.44	14.40 (*BF*_*+0*_)
	c	3.01	.001	.003	0.55	151.52 (*BF*_*+0*_)	0.97	.359	1.00	0.18	0.56 (*BF*_*+0*_)	3.24	< .001	.001	0.59	337.76 (*BF-*_*0*_)
b. LBD																
not impaired gesture imitation	acc	3.30	< .001	.001	0.60	371.72 (*BF*_*-0*_)	3.24	< .001	.001	0.59	193.37 (*BF*_*-0*_)	1.99	.046	.139	0.36	0.21 (*BF*_*0+*_)
	d’	3.07	< .001	.003	0.56	109.91 (*BF*_*-0*_)	2.73	.004	.013	0.50	64.21 (*BF*_*-0*_)	1.70	.095	.284	0.31	0.32 (*BF*_*0+*_)
	c	3.35	< .001	< .001	0.61	302.79 (*BF*_*+0*_)	2.61	.007	.020	0.48	16.56 (*BF*_*+0*_)	1.42	.169	.506	0.26	0.50 (*BF*_*0+*_)
impaired gestures imitation	acc	2.61	.007	.020	0.48	34.11 (*BF*_*-0*_)	1.82	.070	.209	0.33	2.72 (*BF*_*-0*_)	1.90	.057	.172	0.35	0.23 (*BF*_*0+*_)
	d’	2.39	.015	.045	0.44	15.82 (*BF*_*-0*_)	1.25	.229	.688	0.23	1.16 (*BF*_*-0*_)	1.76	.083	.250	0.32	0.49 (*BF*_*0+*_)
	c	2.78	.003	.010	0.51	63.35 (*BF*_*+0*_)	1.36	.188	.563	0.25	1.47 (*BF*_*+0*_)	1.59	.121	.362	0.29	0.65 (*BF*_*0-*_)

*Note*. *p*_*adj*_ *=* Bonferroni adjusted p-values.

*Please note*. Bayes factors *BF*_*+0*_ (acc, d’) and BF_-0_ (c) reflect the support for the alternative hypothesis (better performance during training). Bayes factors *BF*_*0+*_ and *BF*_*0-*_ reflect the support for the null hypothesis (no decline in performance after training). Bayes factors *BF*_*0+*_ and *BF*_*0-*_ < 1 provide support for the alternative hypothesis. Bayes factors *BF*_*+0*_ and *BF*_*-0*_ < 1 provide support for the null hypothesis.

When comparing post training to training performance, the subgroup with impairment in star cancellation performed significantly worse in all three variables when feedback was withdrawn. No significant post training decline in performance was observed for the subgroup without impairment in star cancellation. In contrast, Bayes factors indicate with anecdotal to substantial support that the data are more likely under the assumption of a decline in patients without impairment in star cancellation as well. However, in comparison to the subgroup of patients with impairment in star cancellation (strong to decisive support for a decline), Bayes factors are smaller in patients without impairment in star cancellation.

**Fig 3:**
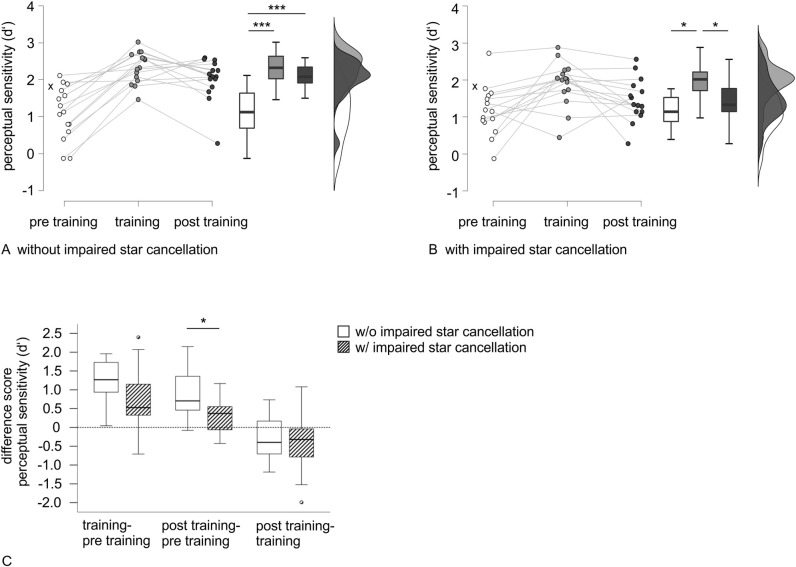
Raincloud plots and boxplots for perceptual sensitivity performance in RBD subgroups. Raincloud plots and significance of timepoint comparisons for perceptual sensitivity performance in the RBD subgroups without (A) and with impairment in star cancellation (B). The cross indicates the median of the healthy control group as a reference. (C) Boxplots for difference scores between timepoints and significance of comparisons between RBD subgroups. Improvement is characterized by values higher than 0, while values below 0 reflect decline. *Note*. * *p* < .05, *** *p* < .001 (after Bonferroni adjustment).

Accordingly, comparing pre training versus post training performance, only the subgroup without impairment in star cancellation performed significantly better post training in all three variables. While the Bayes factor for judgment tendency is in line with this result and provides anecdotal evidence for a lack of pre training to post training difference in the subgroup with impairment in star cancellation, for perceptual sensitivity and accuracy, instead, Bayes factors again indicate anecdotal to substantial evidence for an improvement in these patients as well. However, Bayes factors are larger for patients without impairment in star cancellation and provide decisive evidence for improvement [see 67]. For perceptual sensitivity, raincloud plots for pre training, training and post training performance for the two subgroups are depicted in Fig 3.

Mann-Whitney tests for the difference scores of perceptual sensitivity between the three timepoints (pre training, training, post training) revealed that the subgroups (with vs. without impairment in star cancellation) differed significantly in pre training to post training improvement, demonstrating a higher training gain for patients without impairment in star cancellation (see Table 4 for test statistics). However, no group differences were apparent in pre training to training improvement or training to post training decline. Boxplots for the difference scores are shown in Fig 3C. Bayes factors are in line with these results and provide anecdotal support for no difference between groups in pre training to training improvement and training to post training decline. However, substantial evidence is provided for a difference between groups in pre training to post training gain.

**Table 4:** Mann-Whitney test results comparing difference scores of the three timepoints of measurement between RBD (a.) and LBD (b.) patient subgroups.

**a. RBD**	subgroup w/o vs. subgroup with visuo-spatial deficits														
	training–pre training					post training–pre training					post training–training				
**Variable**	***U***	***p***_***ex***_	***p***_***adj***_	***r***	***BF***_***10***_	***U***	***p***_***ex***_	***p***_***adj***_	***r***	***BF***_***10***_	***U***	***p***_***ex***_	***p***_***adj***_	***r***	***BF***_***10***_
acc	69.50	.076	.227	0.33	0.77	44.50	.004	.012	0.52	6.84	84.50	.245	.735	0.21	0.58
d’	69.00	.074	.223	0.33	0.81	51.50	.010	.031	0.46	4.62	100.00	.624	1.00	0.09	0.42
c	82.00	.217	.651	0.23	0.47	58.50	.024	.073	0.41	2.90	72.00	.098	.293	0.31	1.26
**b. LBD**	subgroup w/o vs. subgroup with motor-cognitive deficits														
	training–pre training					post training–pre training					post training–training				
**Variable**	***U***	***p***_***ex***_	***p***_***adj***_	***r***	***BF***_***10***_	***U***	***p***_***ex***_	***p***_***adj***_	***r***	***BF***_***10***_	***U***	***p***_***ex***_	***p***_***adj***_	***r***	***BF***_***10***_
acc	77.50	.151	.454	0.27	0.82	67.00	.059	.178	0.35	1.29	109.50	.911	1.00	0.02	0.35
d’	76.00	.137	.411	0.28	0.91	70.00	.081	.244	0.32	0.91	107.00	.838	1.00	0.04	0.36
c	85.00	.267	.801	0.21	0.66	92.00	.412	1.00	0.16	0.43	108.00	.870	1.00	0.03	0.35

*Note*. *p*_*adj*_ *=* Bonferroni adjusted p-values.

*Please note*. Bayes factors *BF*_*10*_ reflect the support for the alternative hypothesis (difference between subgroups). A Bayes factor of *BF*_*10*_ < 1 provides support for the null hypothesis (no difference between groups).

The same picture emerged for accuracy. For judgment tendency, there was no significant result in none of the difference scores. The descriptive difference between subgroups in performance gain from pre training to post training did not survive Bonferroni correction. Bayes factors provide anecdotal evidence for no difference between groups in pre training to training improvement but a difference between subgroups in pre training to post training improvement. Anecdotal support is also given for a group difference for training to post training decline in judgment tendency. Patients with impairment in star cancellation demonstrated a larger decline.

#### LBD patients with impairment in hand gesture imitation

Friedman tests revealed a significant difference between timepoints for all three variables for the subgroup without impairment in gesture imitation (perceptual sensitivity: χ^2^(2) = 15.60, *p* < .001; accuracy: χ^2^(2) = 16.14, *p* < .001; judgment tendency: χ^2^(2) = 15.60, *p* < .001) and for perceptual sensitivity and accuracy for the subgroup with impairment in gesture imitation (perceptual sensitivity: χ^2^(2) = 6.53, *p* = .042; accuracy: χ^2^(2) = 9.73, *p* = .007). In the subgroup with impairment in gesture imitation, there was no significant difference between timepoints for judgment tendency (χ^2^(2) = 4.13, *p* = .135). For post-hoc test statistics see Table 3. Both the LBD subgroup without and the LBD subgroup with impairment in gesture imitation showed ameliorated performance in all three variables in the training assessment compared to the pre training assessment. Bayes factors are in accordance with these results and provide very strong to decisive evidence for an improvement during training in the subgroup without impairment in gesture imitation and strong to very strong support for an improvement in the subgroup with impairment in gesture imitation.

Examining training versus post training performance, neither of the subgroups revealed a significant decline in the three variables. Taking Bayes factors into account, there is anecdotal to substantial evidence for a training to post training decline both in patients without and with impairment in gesture imitation.

**Fig 4:**
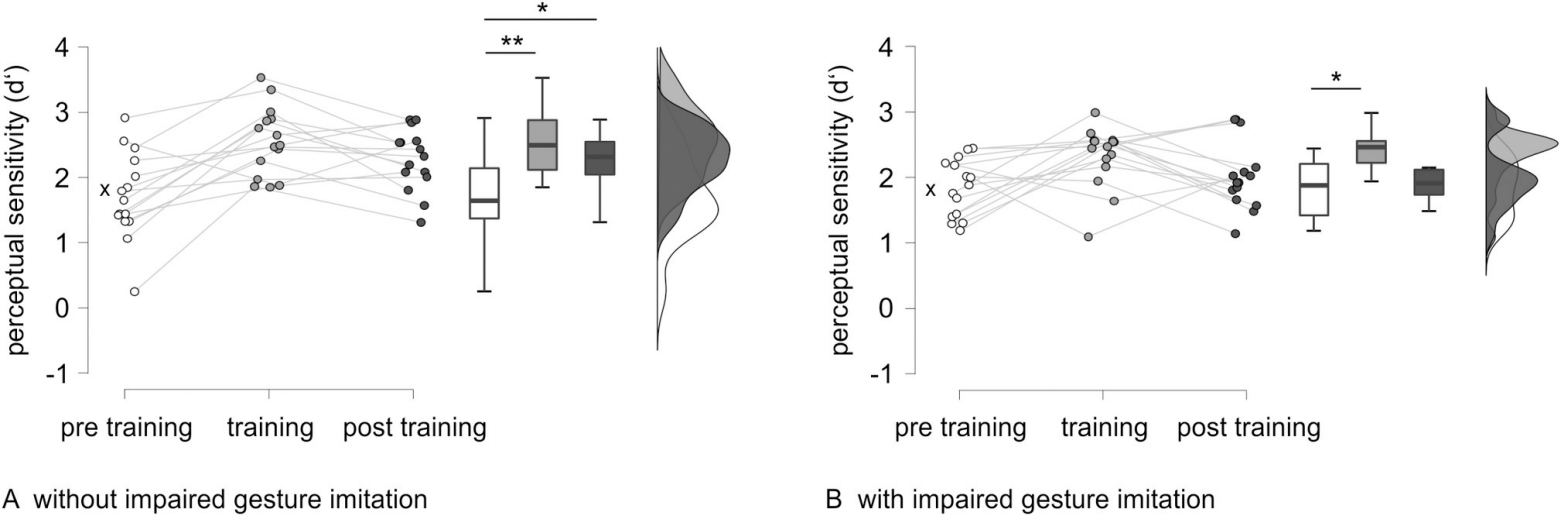
Raincloud plots for perceptual sensitivity performance in the LBD subgroups. Raincloud plots and significance of timepoint comparisons for perceptual sensitivity performance in the LBD subgroups without (A) and with impairment in gesture imitation (B). The cross indicates the median of the healthy control group as a reference. *Note*. * *p* < .05, ** *p* < .01 (after Bonferroni adjustment).

Whereas the subgroup without impairment in gesture imitation improved significantly in perceptual sensitivity performance pre training versus post training, the subgroup with impairment in gesture imitation showed no significant improvement. Bayes factors confirm the improvement in patients without impairment in gesture imitation with strong to decisive support. For the subgroup with impairment in gesture imitation, Bayes factors provide only anecdotal evidence for a pre training to post training improvement in the three variables. Raincloud plots for perceptual sensitivity performance in the two subgroups are depicted in Fig 4.

Mann-Whitney tests for subgroup comparisons of pre training to training improvement, pre training to post training improvement and training to post training decline showed no significant difference between groups (*p*_*adj*_ > .178) in all three variables. Bayes factors reflect anecdotal support for no difference between subgroups in nearly all difference scores of accuracy, perceptual sensitivity and judgment tendency. Only for the pre training to post training improvement in accuracy, there is a Bayes factor greater than 1, indicating anecdotal support for a difference in improvement between groups.

## Discussion

### Aim of the study and main findings

An adequate judgment of action opportunities in relation to the individual’s physical abilities and the environment’s characteristics are important whenever we interact with the environment in everyday life. Stroke patients seem to be at risk for an impairment in so-called affordance judgments (AJs) [21,22]. Further, difficulties in affordance perception of stroke patients are associated with negative consequences for their safety and health [23,24]. In young and older adults, there is evidence that decisions in AJ tasks can be improved by training [7,8]. Based on these findings, the current study examined whether left and right brain damaged stroke patients can improve in affordance perception in an aperture task by means of a short feedback training. In the applied task, patients were invited to judge whether their hand would fit into an aperture with a given opening that varied in width between trials. In addition, we investigated how impairment in a visuo-spatial or motor-cognitive neuropsychological test were associated with trainability in AJs. Overall, we found promising results with improved AJs, when solving the task while the active feedback training was ongoing for all groups. The gain dropped as soon as the intervention stopped, this was particularly the case for patients with impairment in a visuo-spatial task.

Beyond our main analyses, we could replicate aspects of a previous study on affordance judgments in stroke patients of the current study [21]. Whereas most results were in accordance with previous findings of a US sample [21], there were some differences in pre training performance in our sample (see S4 Text for a discussion of similarities and differences between results). Next, we will discuss the main and associated results in light of our study-specific hypotheses.

### Discussion of main and additional results

In support of our overarching assumption (Hypothesis 1), we found that stroke patients can profit from a brief AJs training. Both RBD and LBD patient groups improved in accuracy, perceptual sensitivity and judgment tendency with only a short feedback training session in the Aperture Task. Both groups’ performance increase was obvious during training as well as post training. Our more detailed look at subgroups with deficits in motor-cognitive or visuo-spatial functions supports our hypothesis stating that both patients with and without these deficits improve in accuracy, perceptual sensitivity and judgment tendency during training (Hypothesis 2). While patients with right brain damage and visuo-spatial deficits can profit during the training, this subgroup experienced a performance drop immediately after the training, when the action providing feedback was prohibited. In line with our assumptions (Hypothesis 3), the subgroup with visuo-spatial deficits showed a significant decline in accuracy, perceptual sensitivity and judgment tendency in the post training assessment compared to the training assessment, which was additionally strongly supported by the size of Bayes factors. In the introduction we also stated that in patients with left brain damage and motor-cognitive deficits, we supposed to see a preserved training effect reflected in a post training assessment (Hypothesis 4). Across groups there appeared a training loss when comparing training to post training with no significant difference in training loss between subgroups. Bayes factors indicated evidence for a performance decline when feedback was withdrawn with anecdotal to substantial support. The subgroup with visuo-spatial deficits stood out against the other subgroups by larger Bayes factors with strong evidence for a decline after training.

When comparing pre training to post training improvement patients without impairment in a visuo-spatial or motor cognitive task appear to profit most from training, which is given strong to decisive support by the size of Bayes factors. For the subgroup with visuo-spatial deficits the lack of pre training to post training improvement could be explained by a significant training loss when active feedback is withdrawn during post training testing and a smaller performance gain from pre training to training. Also, in patients with motor-cognitive deficits, a smaller training gain during training may account for a lack of a significantly improved judgment behavior from pre to post training, which is supported by clearly smaller Bayes factors for this subgroup.

As stroke appeared to affect AJs, the trainability is of specific relevance. The novel and encouraging finding of the current study is that stroke patients are trainable in AJs. Also, patients with neuropsychological impairment in a visuo-spatial or motor-cognitive domain were able to adjust their actor-related AJs in the Aperture Task and seemed to profit considerably from the feedback. Reasons need to be unraveled for why there was a significant decline apparent when active feedback was withdrawn in patients with impairment in a visuo-spatial task, particularly in their judgment tendency, and why there was a lack of or only a small training gain pre training to post training, particularly in patients with right brain damage and impairment in a visuo-spatial task as well as in patients with left brain damage and impairment in a motor-cognitive task. One simple reason may be that patients with impairment in a visuo-spatial or motor-cognitive task had difficulties consolidating what had been learned. However, it could also be speculated that heightened attentional processes may play a supportive role for perceiving, encoding and lastly retrieving correct responses. We speculate that both arguments may hold, but the reason for a lack of improvement post training may have to be attributed to a different source, depending on whether patients show impairment in visuo-spatial or motor-cognitive tasks. We first will discuss the results of patients with impairment in the visuo-spatial task and then the results of patients with impairment in the motor-cognitive task.

Interestingly, RBD patients with and without impairment in star cancellation did not differ in pre training performance in the Aperture Task. The disadvantage of patients with impairment in this visuo-spatial task showed itself in reduced gain of training. Thus, the impairment in affordance judgment tasks in patients with impairment in a visuo-spatial task might be less obvious at first sight, but seems to still have significant impact on trainability. We additionally analyzed data from a reaction time task (speeded transport of a dowel) to measure responsiveness in a simple motor task (see S2 Text), reflecting a measure of alertness [68]. The additional finding that reaction times were correlated with performance in RBD patients only during training supports the idea that attentional resources are of specific importance for integrating the gained information during feedback training. We speculate that this patient group profits particularly from acting during the training. These attentional resources confined to action-relevant information are activated in anticipation of the visually guided movement towards the opening that follows the button press as compared to when only pressing a button to indicate the answer. We here would like to refer to the so-called “Enactment effect”. Researchers demonstrated that memory performance (e.g., word lists) was increased when participants were allowed to produce representative actions relative to the verbal task [69–72]. For example, action phrases such as “lift the pen” are recalled better when they are enacted by participants compared to only listened to during verbal tasks. Furthermore, in a doorway passage task with healthy participants, Franchak and colleagues [45] demonstrated that learning by doing improves the accuracy of future judgments about that action compared to a group with judgments based on perception only. Interestingly, a specific benefit of action-relation has been shown in neglect patients [51]. Neglect patients seemed not to be impaired in reaching and finding tasks, especially when the presented objects were defined by their actions as opposed to other properties such as their name or color [50,51]. In the current study, taken together, the significant improvement during training and the significant decline after training in this subgroup could be explained by a distinctive benefit due to the active, target-driven character of the feedback training. On a descriptive level, raincloud plots revealed differential patterns of trainability. Within the group of patients with impairment in a visuo-spatial task, there were two main patterns apparent: First, a triangular pattern with patients who profited considerably from the feedback training with a marked decline after training back to nearly pre training level. Second, a horizontal pattern with patients who seemed to profit only slightly with no strong improvement during training but no decline after training neither. Other than attentional correlates may serve as an explanation. However, with the current data set, we see no possibility to further speculate about the potential mechanisms.

Also, LBD patients showed a significant decline between training and post training assessment in perceptual sensitivity. Against our expectations, the subgroup of LBD patients with impairment in a motor-cognitive task did not improve significantly in pre training to post training comparison. We initially expected a preservation of the training gain, which was based on previous studies that showed trainability of specific motor tasks in patients with impairment in motor-cognitive tasks. However, to our knowledge, trainability of an affordance judgment task comparable to our Aperture Task has not yet been applied in LBD stroke patients. The patient group with impairment in the motor-cognitive task also did not demonstrate any transfer effect to the size estimation task, in contrast to the other patient groups demonstrating improvement. This again is in line with the argument that patients with impairment in a motor-cognitive task may have difficulties learning or consolidating the information gained from the brief training. It needs to be pointed out that the introduced feedback training consisted of only one short session. It is conceivable that patients with impairment in motor-cognitive (or visuo-spatial) tasks need a more intense training in this task in order to achieve enduring improvements, as training intensity has been reported to be an important factor for rehabilitation of motor-cognitive deficits, such as in apraxia [32].

Another argument could be that patients with impaired performance in motor-cognitive tasks have difficulties integrating perceived information and knowledge into an action plan [73,74]. For example, Evans and colleagues [75] suggest that apraxic patients have difficulties integrating visible and known object properties, related to disruptions to the ventro-dorsal stream. In their study, patients with motor-cognitive (apraxic) deficits were asked to grasp novel objects varying in weight distribution. In two conditions, either a memory-associated or a visual-spatial cue indicated the respective object’s weight distribution. Whereas controls adjusted their grasp according to the supplied information, the apraxic patients performed rather poorly in the cued conditions. Potentially, the subgroup with impairment in the motor-cognitive task in the current study could not sustainably integrate the additionally provided information from the feedback training, but rather relied on low-level visual cues instead. In one of our previous studies we were able to demonstrate lack of a stable judgment tendency in LBD patients by implementing a reachability task. We argued that this patient group has a higher reliance on the use of a flexible judgment strategy, using current perceptional information via preserved brain regions [22]. Similarly, Barde and colleagues [76] suggest that patients with motor-cognitive (apraxic) deficits rely disproportionately on object structure (such as size) when learning novel object-related actions. In our current study, the subgroup of patients with impairment in the motor-cognitive task might have relied disproportionately on the visual cue of their hand size, resulting in less training gain during training and similar performance during post training assessment to pre training, where additional visual and haptic information (as supplied during feedback training) was not yet available. Interestingly, however, these patients did show improvement during training. Obviously, it was possible for these patients to partly integrate additional cues when they were allowed to actually try and fit their hand into the opening after each decision. Possibly, the dorsal visual processing stream that is active in on-line computations of the locations of the body and objects [77,78] was deployed during feedback training due to the anticipated action.

This possible shift to the use of the dorsal stream could potentially explain the pattern of better performance during training and a decline after training back to the pre training level in RBD patients with impaired star cancellation. Both patients with impairment in the motor-cognitive task and patients with impairment in the visuo-spatial task with associated lesions in ventro-dorsal areas might have resorted to the dorsal stream, potentially explaining better performance during training. Our reasoning is in line with the results of a meta-analysis by Sakreida and colleagues [79]: This meta-analysis implies that a ventro-dorsal stream reflects an offline mode relying on memorized knowledge, whereas a dorso-dorsal stream reflects an online mode with more direct and dynamic visual information processing.

Our behavioral data appears to support different factors described by the affordance competition model [25], which involves a dynamic interplay of perceptual, attentional, and behavioral biasing processes, contributing to affordance judgments. Brain damage and associated impairment, such as visuo-spatial or motor-cognitive deficits, might lead to interruptions in this complex and dynamic process and add noise to corresponding decision processes. Within this model, feedback training might support impaired affordance judgments on different levels, for example, by supporting the attentional processes through active participation (enactment) and reducing the degrees of freedom by providing information on suitable actions and thereby shaping biases. Our data demonstrated that training advantages differ depending on dysfunctions caused by brain damage. During feedback training, some RBD patients with visuo-spatial deficits might profit from active participation that may bind attention and facilitate processing via the dorsal stream. Post training, this facilitating process is withdrawn, leading to performance reduction. Further, some LBD patients with motor-cognitive deficits might rely disproportionately on low-level visual cues in the competition process, hindering the integration of the gained information during feedback training. Beyond the model that describes a bilateral system, our data including left and right brain damaged patients suggests hemisphere-specific characteristics. The link to more specific neuroanatomical correlates within the affordance competition model needs further investigation, for example, by use of lesion analysis.

### Limitations and outlook

Although not all characteristics of our German sample appeared similar to the sample of a previous study tested in the US [21], we replicated many of our previous findings for the assessment of AJs in an aperture task. This demonstrates the reliability of our results with respect to the paradigm used, but also indicates that the effects of the current training approach should also be tested in different settings.

The current study comes along with the advantages and disadvantages of testing in a clinical setting, where time and space are limited. Even though our results show that feedback training of AJs in stroke patients is promising, the results arose from a specific and controlled task. Therefore, we cannot make a statement about the transferability of the training effect to other AJs. Future studies should consider testing an extended training with respect to sessions and tasks. We demonstrated that already a brief training within one session resulted in an effect of training in AJs in stroke patients. An extended training takes the needs of intensive training for some subgroups into account and could lead to even stronger effects. A longer term follow-up measure should be included to test for sustainability of the training effect. In addition, future studies should disentangle further factors that might have an influence on trainability in affordance judgments, such as general attentional or working memory functions, body representation [80], self awareness [81] or motor imagery capabilities [82], and their relationship to trainings in the investigated sample of stroke patients.

The current study consists of a fundamental research approach. While there are still a lot of questions unanswered within this scope, future studies will nevertheless need to demonstrate its relevance for neurorehabilitation and to the transferability to activities of daily living (ADLs). As trainability of AJs seems to differ depending on the individual deficits, specifically tailored trainings may enhance training effects, especially for patients with impairment in visuo-spatial or motor-cognitive tasks.

### Conclusions

The current study investigated trainability of AJs in patients with right or left hemisphere stroke. In general, trainability could be shown both for patients suffering from right-side brain damage and patients suffering from left-side brain damage. A more detailed analysis of subgroups with and without impairment in a visuo-spatial respectively motor-cognitive task revealed that patients with neuropsychological deficits showed differential profiles of trainability. Both groups seemed to profit considerably from the target-driven action phase during feedback training. Possibly, they relied on an intact dorsal route during training, enabling them to improve in accuracy, perceptual sensitivity and judgment tendency. Bayes factors allowed a more differentiated look beyond classical hypothesis testing and further enabled statements regarding evidential support about hypotheses with no expected differences. Although all patient groups profited from pre to post training as shown by Bayes factors, Bayes factors also demonstrated that they all lost training gain in the post measurement as compared to the training itself. However, it appears that patients with impairment in a visuo-spatial task gain less during training and show greater loss between training and post training.

Overall, it is promising that stroke patients who appeared to be affected in AJ performance can profit from a short training session with active feedback. The differences in trainability for patients with specific neuropsychological deficits, however, should be considered in new training approaches. Lesion analyses could provide more information and allow further discussion.

## Supporting information

S1 Fig Performance in the three pre training blocks of RBD and LBD patients. (DOCX)

S1 Table Demographics and sample characteristics for groups and subgroups. (DOCX)

S2 Table Shapiro-Wilk test results for the residuals by subgroup, variable and timepoint of measurement. (DOCX)

S3 Table Within-subject comparison results (Wilcoxon signed ranks tests) of mere repetition (experimental block Session 1 vs. experimental block Session 2) pre training for the groups and subgroups. (DOCX)

S4 Table Between-subject comparison results (Mann-Whitney tests) for age, Barthel index, time since stroke onset and pre training performance. (DOCX)

S5 Table Correlations (Kendall-Tau b) between time since stroke onset and pre training to post training gain. (DOCX)

S6 Table Comparison between healthy controls who used and judged their right hand vs. healthy controls who used and judged their left hand (Mann-Whitney tests). (DOCX)

S7 Table Within-subject comparison results (paired t-tests) of mere repetition (experimental block Session 1 vs. experimental block Session 2). (DOCX)

S8 Table RmANOVA results per group with impairment in star cancellation (RBD) or impairment in gesture imitation (LBD) as between-subject factor. (DOCX)

S9 Table Post-hoc analyses (paired t-tests) comparing pre training performance with training and post training performance per group. (DOCX)

S10 Table Post-hoc analyses (paired t-tests) comparing pre training performance with training and post training performance per subgroup for RBD (a.) and LBD (b.) patients. (DOCX)

S11 Table Between-subject results (two-sample t-test) comparing difference scores of the three timepoints of measurement between RBD (a.) and LBD (b.) patient subgroups. (DOCX)

S1 Dataset (XLSX)

S1 Text Methods and results of the size estimation control task. (DOCX)

S2 Text Methods and results of the reaction time task. (DOCX)

S3 Text Statistical comparisons of pre training performance between a healthy control group and a patient subsample. (DOCX)

S4 Text Discussion on replications. (DOCX)
