# Supplementary material for: Trainability of affordance judgments in right and left hemisphere stroke patients
Source: PLoS One. 2024 May 3;19(5):e0299705. doi: 10.1371/journal.pone.0299705 (PMC11068188; doi:10.1371/journal.pone.0299705)
Supplement: S1 Fig — (DOCX) [file pone.0299705.s001.docx]

**S4 Fig. Performance in the three pre training blocks of RBD and LBD patients.**

**
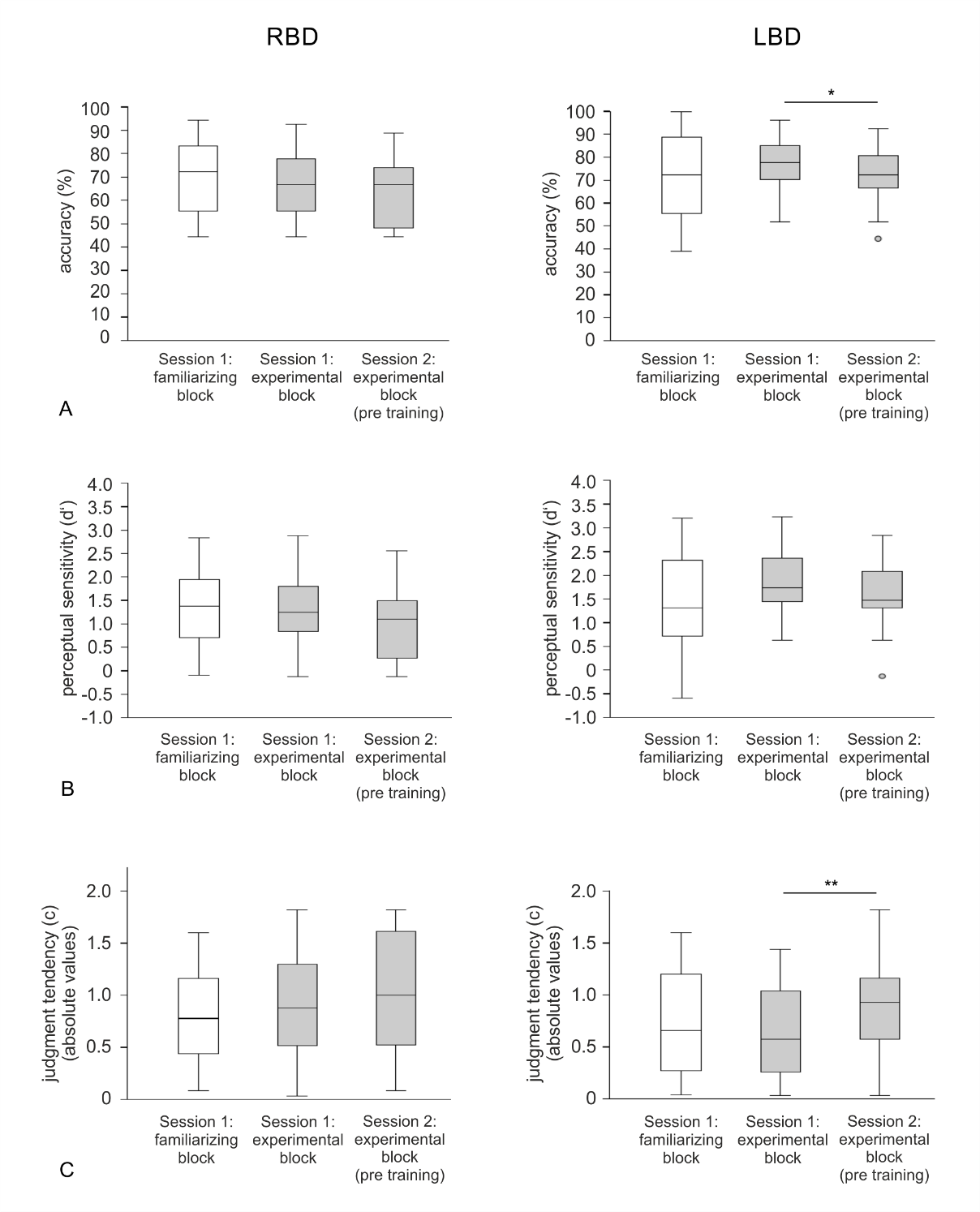
**

*Note.* The familiarizing block consisted of 20 trials, whereas the experimental blocks consisted of 30 trials. The familiarizing block was administered to familiarize the participants with the setting and task, and to allow for the building of a stable response behavior [7]. Therefore, it was not included in statistical analyses. To further analyze effects of mere task exposure or repetition for our experimental trials, we compared the experimental block of Session 1 with the experimental block pre training in Session 2. The data suggests that instead of a repetition benefit, patients rather demonstrate worse performance in Session 2. The difference becomes significant in LBD patients.

Furthermore, for our study purposes the experimental blocks of the first session and the pre training were merged to one pre training measure. This was done to calculate with a more representative measure.

*Please note.* * *p* < .05, ** *p* < .01
