# Supplementary material for: Trainability of affordance judgments in right and left hemisphere stroke patients
Source: PLoS One. 2024 May 3;19(5):e0299705. doi: 10.1371/journal.pone.0299705 (PMC11068188; doi:10.1371/journal.pone.0299705)
Supplement: S1 Table — (DOCX) [file pone.0299705.s002.docx]

# **S1 Table. Demographics and sample characteristics for groups and subgroups.**

|  | age | gender | Barthel index | DSO | hand imitation score | hand imitation below cut-off | star cancellation score | star cancellation below cut-off | star cancellation LUSN |
| --- | --- | --- | --- | --- | --- | --- | --- | --- | --- |
| **Group** | ***M* (*SD*)** | ***n*** | ***M* (*SD*)** | ***M* (*SD*)** | ***M* (*SD*)** | ***n*** | ***M* (*SD*)** | ***n*** | ***n*** |
| a. RBD | 60.03 (12.10) | female: 12 male: 18 | 53.46 (18.91) | 90 (70) | 15.83 (3.63) | no: 24 yes: 6 | 46.13  (10.52) | no: 15 yes: 15 | no: 20 yes: 10 |
| not impaired star cancellation | 59.07 (14.74) | female: 7 male: 8 | 58.85 (20.93) | 62 (29) | 16.40 (3.58) | no: 13 yes: 2 | 53.20 (0.78) | no: 15 yes: 0 | no: 15 yes: 0 |
| impaired star cancellation | 61.00 (9.17) | female: 5 male: 10 | 48.08 (15.62) | 118 (88) | 15.27 (3.71) | no: 11 yes: 4 | 39.07 (11.02) | no: 0 yes: 15 | no: 5 yes: 10 |
| b. LBD | 62.13 (13.06) | female: 13 male: 17 | 55.00 (16.69) | 67 (37) | 14.37 (4.72) | no: 15 yes: 15 | 51.87 (4.62) | no: 26 yes: 4 | no: 30 yes: 0 |
| not impaired gesture imitation | 60.33 (12.93) | female: 7 male: 8 | 56.07 (14.70) | 54 (20) | 17.93 (1.44) | no: 15 yes: 0 | 51.87 (6.40) | no: 14 yes: 1 | no: 15 yes: 0 |
| impaired gesture imitation | 63.93 (13.39) | female: 6 male: 9 | 54.00 (18.82) | 81 (45) | 10.80 (4.09) | no: 0 yes: 15 | 51.87 (1.81) | no: 12 yes: 3 | no: 15 yes: 0 |

*Note.* LUSN = left unilateral spatial neglect; DSO = days since stroke onset. In the RBD group, for two patients with and for two patients without impairment in star cancellation no Barthel index was available. In the LBD group, for one patient without impairment in gesture imitation no Barthel index was available.
