# Supplementary material for: Trainability of affordance judgments in right and left hemisphere stroke patients
Source: PLoS One. 2024 May 3;19(5):e0299705. doi: 10.1371/journal.pone.0299705 (PMC11068188; doi:10.1371/journal.pone.0299705)
Supplement: S2 Table — (DOCX) [file pone.0299705.s003.docx]

# **S2 Table. Shapiro-Wilk test results for the residuals by subgroup, variable and timepoint of measurement.**

|  |  |  | pre training | | | training | | | post training | | | |
| --- | --- | --- | --- | --- | --- | --- | --- | --- | --- | --- | --- | --- |
| Group |  | Variable | Stat. | df | Sig. | Stat. | df | Sig. | Stat. | df | Sig. |  |
| RBD | not impaired star cancellation | acc | .958 | 15 | .663 | .853 | 15 | .019 | .730 | 15 | <.001 |  |
|  |  | d’ | .951 | 15 | .539 | .971 | 15 | .870 | .799 | 15 | .004 |  |
|  |  | c | .953 | 15 | .576 | .892 | 15 | .073 | .850 | 15 | .018 |  |
|  | impaired star cancellation | acc | .954 | 15 | .585 | .876 | 15 | .042 | .976 | 15 | .937 |  |
|  |  | d’ | .968 | 15 | .827 | .929 | 15 | .260 | .981 | 15 | .977 |  |
|  |  | c | .887 | 15 | .060 | .876 | 15 | .041 | .936 | 15 | .331 |  |
| LBD | not impaired gesture immitation | acc | .952 | 15 | .557 | .949 | 15 | .502 | .855 | 15 | .021 |  |
|  |  | d’ | .969 | 15 | .837 | .946 | 15 | .470 | .951 | 15 | .541 |  |
|  |  | c | .958 | 15 | .665 | .923 | 15 | .212 | .940 | 15 | .380 |  |
|  | impaired gesture imitation | acc | .932 | 15 | .288 | .846 | 15 | .015 | .909 | 15 | .129 |  |
|  |  | d’ | .928 | 15 | .253 | .854 | 15 | .020 | .909 | 15 | .131 |  |
|  |  | c | .954 | 15 | .595 | .889 | 15 | .065 | .887 | 15 | .061 |  |
