# Supplementary material for: Trainability of affordance judgments in right and left hemisphere stroke patients
Source: PLoS One. 2024 May 3;19(5):e0299705. doi: 10.1371/journal.pone.0299705 (PMC11068188; doi:10.1371/journal.pone.0299705)
Supplement: S3 Table — (DOCX) [file pone.0299705.s004.docx]

S3 Table. Within-subject comparison results (Wilcoxon signed ranks tests) of mere repetition (experimental block Session 1 vs. experimental block Session 2) pre training for the groups and subgroups.

| a. | RBD | | | not impaired star cancellation | | | impaired star cancellation | | |
| --- | --- | --- | --- | --- | --- | --- | --- | --- | --- |
| Variable | *z* | *p* | *BF* | *z* | *p* | *BF* | *z* | *p* | *BF* |
| accuracy | 1.16 | .254 | 8.92  *(BF_0-_)* | 1.06 | .314 | 5.82  *(BF_0-_)* | 0.44 | .688 | 5.14  *(BF_0-_)* |
| perceptual sensitivity (d’) | 1.12 | .271 | 10.99  *(BF_0-_)* | 1.10 | .292 | 7.63  *(BF_0-_)* | 0.41 | .715 | 5.40  *(BF_0-_)* |
| judgment tendency (c) | 1.51 | .134 | 11.55  (*BF_0+_*) | 0.94 | .370 | 6.41  (*BF_0+_*) | 1.10 | .296 | 7.07  (*BF_0+_*) |
| b. | LBD | | | not impaired gesture imitation | | | impaired gesture imitation | | |
| Variable | *z* | *p* | *BF* | *z* | *p* | *BF_0-_* | *z* | *p* | *BF_0-_* |
| accuracy | 2.14 | .032 | 14.07  *(BF_0-_)* | 0.79 | .451 | 6.22  *(BF_0-_)* | 2.41 | .014 | 12.33  *(BF_0-_)* |
| perceptual sensitivity (d’) | 1.78 | .076 | 12.74  *(BF_0-_)* | 0.97 | .349 | 7.51  *(BF_0-_)* | 1.48 | .153 | 8.55  *(BF_0-_)* |
| judgment tendency (c) | 2.96 | .002 | 18.65  (*BF_0+_*) | 1.29 | .211 | 7.96  (*BF_0+_*) | 3.05 | <.001 | 13.72  (*BF_0+_*) |

*Note.* The significant results in LBD patients reflect a decline in performance at repetition of the task. *Please note.* BF_0-_ reflects an alternative hypothesis of higher (= better) values in the second session (accuracy and perceptual sensitivity). As lower scores of judgment tendency describe better performance, the alternative hypothesis is opposite (BF_0+_) for this variable. Both Bayes factors reflect the support for the null hypothesis (no improvement due to mere repetition).
