# Supplementary material for: Trainability of affordance judgments in right and left hemisphere stroke patients
Source: PLoS One. 2024 May 3;19(5):e0299705. doi: 10.1371/journal.pone.0299705 (PMC11068188; doi:10.1371/journal.pone.0299705)
Supplement: S4 Table — (DOCX) [file pone.0299705.s005.docx]

**S5 Table. Between-subject comparison results (Mann-Whitney tests) for age, Barthel index, time since stroke onset and pre training performance.**

|  | RBD vs. LBD | | | impaired vs. not impaired star cancellation | | | impaired vs. not impaired gesture imitation | | |
| --- | --- | --- | --- | --- | --- | --- | --- | --- | --- |
| Variable | *U* | *p* | *BF_01_* | *U* | *p* | *BF_01_* | *U* | *p* | *BF_01_* |
| Age | 407.50 | .535 | 2.65 | 107.50 | .846 | 2.81 | 96.50 | .518 | 2.83 |
| Barthel index | 337.00 | .503 | 3.33 | 56.00 | .148 | 1.32 | 98.50 | .787 | 2.69 |
| days since stroke onset | 355.50 | .165 | 1.73 | 52.00 | .011 | 0.33 | 78.50 | .163 | 1.22 |
| perceptual sensitivity (d’) | 204.50 | <.001 | 0.04 | 110.50 | .943 | 2.83 | 105.00 | .766 | 2.88 |
| accuracy (%) | 238.00 | .001 | 0.08 | 96.50 | .518 | 2.54 | 97.50 | .545 | 2.71 |
| judgment tendency (c) | 346.50 | .128 | 1.08 | 78.50 | .164 | 1.48 | 97.00 | .532 | 2.27 |

*Note.* Bayes factors *BF_01_* reflect the support for the null hypothesis (no difference between groups). Bayes factors (*BF_01_*) < 1 provide support for the alternative hypothesis (difference between groups).
