# Supplementary material for: Trainability of affordance judgments in right and left hemisphere stroke patients
Source: PLoS One. 2024 May 3;19(5):e0299705. doi: 10.1371/journal.pone.0299705 (PMC11068188; doi:10.1371/journal.pone.0299705)
Supplement: S5 Table — (DOCX) [file pone.0299705.s006.docx]

**S6 Table. Correlations (Kendall-Tau b) between time since stroke onset and pre training to post training gain.**

|  |  |  | **accuracy** difference score (post training-pre training) | **d’** difference score (post training-pre training) | **c** difference score (post training-pre training) |
| --- | --- | --- | --- | --- | --- |
| whole sample | time since stroke onset (days) | correlation coefficient (τ_b_) | -.044 | -.019 | .015 |
|  |  | Sig. (2-tailed) | .623 | .833 | .863 |
|  |  | *n* | 60 | 60 | 60 |
| RBD | time since stroke onset (days) | correlation coefficient (τ_b_) | -.165 | -.175 | .092 |
|  |  | Sig. (2-tailed) | .205 | .175 | .475 |
|  |  | *n* | 30 | 30 | 30 |
| LBD | time since stroke onset (days) | correlation coefficient (τ_b_) | .094 | .107 | -.079 |
|  |  | Sig. (2-tailed) | .474 | .411 | .543 |
|  |  | *n* | 30 | 30 | 30 |
