# Supplementary material for: Trainability of affordance judgments in right and left hemisphere stroke patients
Source: PLoS One. 2024 May 3;19(5):e0299705. doi: 10.1371/journal.pone.0299705 (PMC11068188; doi:10.1371/journal.pone.0299705)
Supplement: S6 Table — (DOCX) [file pone.0299705.s007.docx]

S10 Table. Comparison between healthy controls who used and judged their right hand vs. healthy controls who used and judged their left hand (Mann-Whitney tests).

|  | accuracy (%) | perceptual sensitivity (d’) | | judgment tendency (c) | |
| --- | --- | --- | --- | --- | --- |
| *U* | 23.00 | | 24.50 | | 21.50 |
| *p_ex_* | .666 | | .799 | | .549 |
| *BF_01_* | 1.92 | | 2.02 | | 2.00 |

*Note.* Bayes factors *BF_01_* > 1 reflect support for the null hypothesis (no difference between groups).
