# Supplementary material for: Trainability of affordance judgments in right and left hemisphere stroke patients
Source: PLoS One. 2024 May 3;19(5):e0299705. doi: 10.1371/journal.pone.0299705 (PMC11068188; doi:10.1371/journal.pone.0299705)
Supplement: S7 Table — (DOCX) [file pone.0299705.s008.docx]

**S12 Table.** **Within-subject comparison results (paired t-tests) of mere repetition (experimental block Session 1 vs. experimental block Session 2).**

| a. | RBD | | | not impaired star cancellation | | | impaired star cancellation | | |
| --- | --- | --- | --- | --- | --- | --- | --- | --- | --- |
| Variable | *t* | df | *p* | *t* | df | *p* | *t* | df | *p* |
| accuracy | 1.08 | 29 | .288 | 1.04 | 14 | .315 | 0.44 | 14 | .667 |
| perceptual sensitivity (d’) | 1.10 | 29 | .279 | 1.10 | 14 | .290 | 0.45 | 14 | .658 |
| judgment tendency (c) | 1.52 | 29 | .139 | 0.99 | 14 | .337 | 1.14 | 14 | .273 |
| b. | LBD | | | not impaired gesture imitation | | | impaired gesture imitation | | |
| Variable | *t* | df | *p* | *t* | df | *p* | *t* | df | *p* |
| accuracy | 2.36 | 29 | .025 | 0.86 | 14 | .406 | 2.86 | 14 | .013 |
| perceptual sensitivity (d’) | 1.84 | 29 | .076 | 0.90 | 14 | .381 | 1.79 | 14 | .096 |
| judgment tendency (c) | 3.18 | 29 | .003 | 1.22 | 14 | .242 | 4.42 | 14 | <.001 |

*Note.* The significant results in LBD patients reflect a decline in performance at repetition of the task.

*Please note.* There are no deviations in significance compared to the non-parametric analyses.
