# Supplementary material for: Trainability of affordance judgments in right and left hemisphere stroke patients
Source: PLoS One. 2024 May 3;19(5):e0299705. doi: 10.1371/journal.pone.0299705 (PMC11068188; doi:10.1371/journal.pone.0299705)
Supplement: S8 Table — (DOCX) [file pone.0299705.s009.docx]

**S13 Table.** **RmANOVA results per group with impairment in star cancellation (RBD) or impairment in gesture imitation (LBD) as between-subject factor.**

|  | accuracy (%) | | | | | perceptual sensitivity (d’) | | | | judgment tendency (c) | | | | |
| --- | --- | --- | --- | --- | --- | --- | --- | --- | --- | --- | --- | --- | --- | --- |
| **Group** | *F* | df | df error | *p* | *F* | | df | df error | *p* | *F* | df | df error | *p* |  |
| RBD |  |  |  |  |  | |  |  |  |  |  |  |  |  |
| timepoint | 34.84 | 2 | 56 | <.001 | 33.78 | | 2 | 56 | <.001 | 25.77 | 2 | 56 | <.001 |  |
| timepoint * impairment in star cancellation | 4.44 | 2 | 56 | .016 | 3.64 | | 2 | 56 | .033 | 4.13 | 2 | 56 | .021 |  |
| impairment in star cancellation | 1.10 | 1 | 28 | .304 | 3.27 | | 1 | 28 | .081 | 0.58 | 1 | 28 | .452 |  |
| LBD |  |  |  |  |  | |  |  |  |  |  |  |  |  |
| timepoint | 25.15 | 2 | 56 | <.001 | 17.45 | | 2 | 56 | <.001 | 16.73 | 2 | 56 | <.001 |  |
| timepoint * impairment in gesture imitation | 2.43 | 2 | 56 | .097 | 1.76 | | 2 | 56 | .181 | 1.01 | 2 | 56 | .371 |  |
| impairment in gesture imitation | 0.23 | 1 | 28 | .634 | 0.78 | | 1 | 28 | .386 | 0.28 | 1 | 28 | .599 |  |
