# Supplementary material for: Trainability of affordance judgments in right and left hemisphere stroke patients
Source: PLoS One. 2024 May 3;19(5):e0299705. doi: 10.1371/journal.pone.0299705 (PMC11068188; doi:10.1371/journal.pone.0299705)
Supplement: S9 Table — (DOCX) [file pone.0299705.s010.docx]

**S14 Table**. **Post-hoc analyses (paired t-tests) comparing pre training performance with training and post training performance per group.**

|  |  | pre training vs. training | | | | | pre training vs. post training | | | | | | training vs. post training | | | | |
| --- | --- | --- | --- | --- | --- | --- | --- | --- | --- | --- | --- | --- | --- | --- | --- | --- | --- |
| **Group** | **Variable** | *t* | df | ***p_ex_*** | ***p_adj_*** | ***d*** | *t* | df | ***p_ex_*** | ***p_adj_*** | ***d*** | *t* | | df | ***p_ex_*** | ***p_adj_*** | ***d*** |
| RBD | acc | 7.17 | 29 | <.001 | <.001 | 1.31 | 5.03 | 29 | <.001 | <.001 | 0.92 | 3.08 | | 29 | .004 | .013 | 0.56 |
|  | d’ | 7.09 | 29 | <.001 | <.001 | 1.29 | 5.09 | 29 | <.001 | <.001 | 0.93 | 3.17 | | 29 | .004 | .011 | 0.58 |
|  | c | 6.58 | 29 | <.001 | <.001 | 1.20 | 3.24 | 29 | .003 | .009 | 0.59 | 3.86 | | 29 | .001 | .002 | 0.71 |
| LBD | acc | 6.23 | 29 | <.001 | <.001 | 1.14 | 4.17 | 29 | <.001 | .001 | 0.76 | 2.97 | | 29 | .006 | .018 | 0.54 |
|  | d’ | 5.18 | 29 | <.001 | <.001 | 0.95 | 3.43 | 29 | .002 | .005 | 0.63 | 2.81 | | 29 | .009 | .027 | 0.51 |
|  | c | 6.08 | 29 | <.001 | <.001 | 1.11 | 3.13 | 29 | .004 | .012 | 0.57 | 2.38 | | 29 | .024 | .072 | 0.44 |

*Note.* *p_adj_* = Bonferroni adjusted p-values.

*Please note.* There are no deviations in significance compared to the non-parametric analyses.
