# Supplementary material for: Trainability of affordance judgments in right and left hemisphere stroke patients
Source: PLoS One. 2024 May 3;19(5):e0299705. doi: 10.1371/journal.pone.0299705 (PMC11068188; doi:10.1371/journal.pone.0299705)
Supplement: S10 Table — (DOCX) [file pone.0299705.s011.docx]

**S15 Table. Post-hoc analyses (paired t-tests) comparing pre training performance with training and post training performance per subgroup for RBD (a.) and LBD (b.) patients.**

|  |  | pre training vs. training | | | | | pre training vs. post training | | | | | training vs. post training | | | | |
| --- | --- | --- | --- | --- | --- | --- | --- | --- | --- | --- | --- | --- | --- | --- | --- | --- |
| **Group** | **Var.** | *t* | df | ***p_ex_*** | ***p_adj_*** | ***d*** | *t* | df | ***p_ex_*** | ***p_adj_*** | ***d*** | *t* | df | ***p_ex_*** | ***p_adj_*** | ***d*** |
| a. RBD |  |  |  |  |  |  |  |  |  |  |  |  |  |  |  |  |
| not impaired star cancellation | Acc | 7.74 | 14 | <.001 | <.001 | 2.00 | 6.00 | 14 | <.001 | <.001 | 1.55 | 1.83 | 14 | .089 | .267 | 0.47 |
|  | d’ | 7.92 | 14 | <.001 | <.001 | 2.05 | 5.38 | 14 | <.001 | <.001 | 1.39 | 2.10 | 14 | .055 | .164 | 0.54 |
|  | c | 5.61 | 14 | <.001 | <.001 | 1.45 | 4.68 | 14 | <.001 | .001 | 1.21 | 1.76 | 14 | .100 | .301 | 0.45 |
| impaired star cancellation | Acc | 3.53 | 14 | .003 | .010 | 0.91 | 1.94 | 14 | .073 | .219 | 0.50 | 2.51 | 14 | .025 | **.075** | 0.65 |
|  | d’ | 3.40 | 14 | .004 | .013 | 0.88 | 2.34 | 14 | .034 | .103 | 0.61 | 2.33 | 14 | .035 | **.105** | 0.60 |
|  | c | 3.79 | 14 | .002 | .006 | 0.98 | 0.59 | 14 | .562 | 1.00 | 0.15 | 3.74 | 14 | .002 | .007 | 0.96 |
| b. LBD |  |  |  |  |  |  |  |  |  |  |  |  |  |  |  |  |
| not impaired gesture imitation | Acc | 5.48 | 14 | <.001 | <.001 | 1.41 | 4.87 | 14 | <.001 | .001 | 1.26 | 2.15 | 14 | **.050** | .149 | 0.56 |
|  | d’ | 4.87 | 14 | <.001 | .001 | 1.26 | 3.81 | 14 | .002 | .006 | 0.98 | 2.07 | 14 | .058 | .173 | 0.53 |
|  | c | 4.92 | 14 | <.001 | .001 | 1.27 | 3.24 | 14 | .006 | .018 | 0.84 | 1.79 | 14 | .095 | .285 | 0.46 |
| impaired gesture imitation | Acc | 3.51 | 14 | .003 | .010 | 0.91 | 1.58 | 14 | .136 | .407 | 0.41 | 2.03 | 14 | .062 | .187 | 0.52 |
|  | d’ | 2.65 | 14 | .019 | **.057** | 0.68 | 1.23 | 14 | .237 | .712 | 0.32 | 1.87 | 14 | .083 | .249 | 0.48 |
|  | c | 3.77 | 14 | .002 | .006 | 0.97 | 1.41 | 14 | .181 | .542 | 0.36 | 1.51 | 14 | .152 | .457 | 0.39 |

*Note.* *p_adj_* = Bonferroni adjusted p-values.

*Please note.* Deviations in significance compared to the non-parametric analyses are bold printed.
