# Supplementary material for: Trainability of affordance judgments in right and left hemisphere stroke patients
Source: PLoS One. 2024 May 3;19(5):e0299705. doi: 10.1371/journal.pone.0299705 (PMC11068188; doi:10.1371/journal.pone.0299705)
Supplement: S11 Table — (DOCX) [file pone.0299705.s012.docx]

**S16 Table. Between-subject results (two-sample t-test) comparing difference scores of the three timepoints of measurement between RBD (a.) and LBD (b.) patient subgroups.**

| **a.** **RBD** | not impaired vs. impaired subgroup (star cancellation) | | | | | | | | | | | | | | | | |  |
| --- | --- | --- | --- | --- | --- | --- | --- | --- | --- | --- | --- | --- | --- | --- | --- | --- | --- | --- |
|  | difference score training – pre training | | | | | | difference score post training – pre training | | | | | difference score  post training – training | | | | | |  |
| **Variable** | | *t* | df | ***p_ex_*** | ***p_adj_*** | ***d*** | *t* | df | ***p_ex_*** | ***p_adj_*** | ***d*** | *t* | df | ***p_ex_*** | ***p_adj_*** | ***d*** |  |  |
| Acc | | 1.65 | 28 | .110 | .329 | 0.60 | 3.26 | 28 | .003 | .009 | 1.19 | 1.14 | 28 | .262 | .787 | 0.42 |  |  |
| d’ | | 1.82 | 28 | .080 | .241 | 0.66 | 2.92 | 28 | .007 | .020 | 1.07 | 0.55 | 28 | .585 | 1.00 | 0.20 |  |  |
| c | | 1.08 | 28 | .289 | .867 | 0.40 | 2.69 | 28 | .012 | **.036** | 0.98 | 2.02 | 28 | .053 | .158 | 0.74 |  |  |
| **b.** **LBD** | | not impaired vs. impaired subgroup (gesture imitation) | | | | | | | | | | | | | | | | |
|  | | difference score training – pre training | | | | | difference score post training – pre training | | | | | difference score  post training – training | | | | | | |
| **Variable** | | *t* | df | ***p_ex_*** | ***p_adj_*** | ***d*** | *t* | df | ***p_ex_*** | ***p_adj_*** | ***d*** | *t* | df | ***p_ex_*** | ***p_adj_*** | ***d*** |  |  |
| Acc | | 1.64 | 28 | .112 | .336 | 0.60 | 1.98 | 28 | .058 | .173 | 0.72 | 0.24 | 28 | .812 | 1.00 | 0.09 |  |  |
| d’ | | 1.40 | 28 | .172 | .515 | 0.51 | 1.83 | 28 | .078 | .235 | 0.67 | 0.11 | 28 | .913 | 1.00 | 0.04 |  |  |
| c | | 1.46 | 28 | .157 | .470 | 0.53 | 0.93 | 28 | .359 | 1.00 | 0.34 | 0.30 | 28 | .766 | 1.00 | 0.11 |  |  |

*Note.* *p_adj_* = Bonferroni adjusted p-values.

*Please note.* Deviations in significance compared to the non-parametric analyses are bold printed.
