# Supplementary material for: Trainability of affordance judgments in right and left hemisphere stroke patients
Source: PLoS One. 2024 May 3;19(5):e0299705. doi: 10.1371/journal.pone.0299705 (PMC11068188; doi:10.1371/journal.pone.0299705)
Supplement: S1 Text — (DOCX) [file pone.0299705.s014.docx]

**S7 Text. Methods and Results of the size estimation control task.**

Size estimation task: Methods

The size estimation task served as control task to assess the participants’ ability to estimate their hands’ horizontal size. The opening’s width of the apparatus was gradually increased or decreased and the participants were instructed to verbally indicate when the opening width reflected the size of their hand’s widest part. Participants were allowed to verbally indicate readjustments of the chosen horizontal opening until they thought they had the best possible estimation. The vertical width was not changed and corresponded to the participant’s actual vertical hand size. In total, 16 trials of the task were completed, half of the trials starting from a 200 mm opening and half of the trials from 0 mm. Whether the left or the right hand served as stimulus varied in a fixed randomized order and the start opening alternated each trial. As in the main task, participants could see their hand on the table in the same fixed and marked positions (see Figure 1). Participants did not receive feedback for the task and were not allowed to reach out their arms and check their estimations.

Size estimation task: Results

One LBD patient did not complete the size estimation task and therefore was excluded from size estimation task analysis. When participants stopped the gradually opening or closing aperture at their estimated hand’s size, an exact Mann-Whitney test revealed that LBD and RBD patients differed significantly only before training in their mean deviation from their actual hand size (*U* = 285.50, *p* = .023, *r* = 0.30, *BF_10_ =* 1.83), but not after training (*U* = 359.50, *p* = .256, r = 0.15, *BF_10_ =* 0.38). Before training, the RBD patient group showed a significantly higher deviation from the actual hand size (RBD: *M_dn_* = 27.75 mm, LBD: *M_dn_* = 12.50 mm).

Neither before nor after training, the RBD subgroup with impairment in star cancellation differed significantly from the RBD subgroup without impairment in star cancellation (before training: *U* = 94.50, *p* = .467, *r* = 0.14, *BF_10_ =* 0.44; after training: *U* = 97.00, *p* = .539, *r* = 0.12,
*BF_10_ =* 0.38). Neither did the LBD subgroup with impairment in gesture imitation and the LBD subgroup without impairment in gesture imitation differ in the size estimation deviation (before training: *U* = 82.50, *p* = .337, *r* = 0.18, *BF_10_* = 0.63; after training: *U* = 105,00, *p* = 1.00,
*r* = 0.00, *BF_10_ =* 0.34). Both the RBD subgroup without (*z* = 2.00, *p* = .046, *r* = 0.36,
*BF_10_* = 1.69) and with impairment in star cancellation (*z* = 2.84, *p* = .002, *r* = 0.52, *BF_10_* = 46.51) estimated their hand’s size in the size estimation task better in the second session (after training). In LBD patients, only the subgroup without impairment in gesture imitation (*z* = 2.01, *p* = .044, *r* = 0.37, *BF_10_* = 2.08) showed improved hand size estimation in the second session (with impairment in gesture imitation: *z* = 0.28, *p* = .796, *r* = 0.05, *BF_10_* = 0.27). After training, the deviation in size estimation of RBD patients (*M_dn_* = 17.75 mm) was similar to the deviation in size estimation of the healthy control group before training (*M_dn_* = 17.50 mm).
