# Supplementary material for: Trainability of affordance judgments in right and left hemisphere stroke patients
Source: PLoS One. 2024 May 3;19(5):e0299705. doi: 10.1371/journal.pone.0299705 (PMC11068188; doi:10.1371/journal.pone.0299705)
Supplement: S2 Text — (DOCX) [file pone.0299705.s015.docx]

**S8 Text. Methods and Results of the reaction time task.**

Reaction time task: Methods

A reaction time task served as an additional measure to enable the examination of a correlation between alertness and performance in the AJ task. The reaction time measure was part of the neuropsychological test battery administered before the two consecutive AJ task sessions. The task was to transport a bar on a rack, either upward or downward, alternating between the two different levels as quickly as possible. Participants produced one transport movement per trial. The task consisted of 16 trials. As patients needed to get familiar with the procedure, the first four trials were excluded from analysis. Participants were instructed to hold a key until Plato goggles opened. Participants transported a horizontal bar into the respective available level of the rack. The time between the opening of the Plato goggles and the release of the keypress served as reaction time measure. The median of reaction times was calculated from the remaining 12 trials.

**Reaction time task: Data analysis**

Correlations (Kendall’s tau) were calculated between patients’ reaction times (median) and the variables accuracy and perceptual sensitivity, for LBD and RBD patients respectively.

**Reaction time task: Results**

One LBD patient did not take part in the reaction time task and therefore was not included in reaction time analysis.

In RBD patients, there was no significant correlation between reaction times and pre training accuracy (τ_b_ = -.14, *p* = .275) or perceptual sensitivity (τ_b_ = -.16, *p* = .231). During training, a correlation significantly different from zero between reaction times and both accuracy (τ_b_ = -.29, *p* = .025) and perceptual sensitivity (τ_b_ = -.30, *p* = .020) was apparent. Slower reaction times went along with lower performance in the AJ task during training.

In LBD patients, there was no significant correlation between reaction times and accuracy or perceptual sensitivity, neither pre training (accuracy: τ_b_ = .16, *p* = .229; perceptual sensitivity: τ_b_ = .10, *p* = .442) nor during training (accuracy: τ_b_ = -.21, *p* = .122; perceptual sensitivity: τ_b_ = -.16, *p* = .223).
