# Supplementary material for: Trainability of affordance judgments in right and left hemisphere stroke patients
Source: PLoS One. 2024 May 3;19(5):e0299705. doi: 10.1371/journal.pone.0299705 (PMC11068188; doi:10.1371/journal.pone.0299705)
Supplement: S3 Text — (DOCX) [file pone.0299705.s016.docx]

**S9 Text. Statistical comparisons of pre training performance between a healthy control group and a patient subsample.**

Healthy controls (N = 15) were matched to patients (N = 15) with respect to hand used and judged for (9 right, 6 left), gender (4 female, 11 male), and age (healthy controls: *M* = 55.27, *SD* = 14.92; patients: *M* = 55,73, *SD* = 14.27).

To test for worse performance in the AJ task in the stroke patient group compared to healthy controls, we ran one-tailed Mann-Whitney tests.

Comparing patients’ AJs pre training performance to healthy controls one-tailed, stroke patients showed lower accuracy (*U* = 69.50, *p* = .038, *r* = 0.33, *BF_+0_* = 1.94) and perceptual sensitivity (*U* = 68.50, *p* = .035, *r* = 0.33, *BF_+0_* = 1.75). Bayes factors provide anecdotal support. There was not a significantly worse performance in patients with regard to judgment tendency (*U* = 90.50, *p* = .186, *r* = 0.17, *BF_-0_* = 0.92), which is supported by a Bayes factor below 1, indicating rather support for no difference between healthy controls and patients with regard to judgment tendency.
