# Supplementary material for: Trainability of affordance judgments in right and left hemisphere stroke patients
Source: PLoS One. 2024 May 3;19(5):e0299705. doi: 10.1371/journal.pone.0299705 (PMC11068188; doi:10.1371/journal.pone.0299705)
Supplement: S4 Text — (DOCX) [file pone.0299705.s017.docx]

**S11 Text. Discussion on Replications.**

The current study results are in line with a prior study reporting data from a US sample showing that patients suffering from stroke perform significantly worse compared to healthy controls in judging whether their hand could fit into a given opening [1]. Also in accordance with the findings by Randerath and colleagues [1], we did not find a significant difference in judgment tendency between patients and healthy controls. A few differences in our sample characteristics compared to Randerath and colleagues [1] emerged for pre training performance. Descriptively, the healthy control groups of both studies showed a very similar median in perceptual sensitivity, but our current LBD patient group showed a slightly better performance, whereas our RBD patient group showed worse performance. As a result and in contrast to the previous findings, the comparison of our current LBD versus RBD patient groups demonstrated a significant difference in pre training performance, with RBD patients performing worse. Further, Randerath and colleagues [1] found significantly lower perceptual sensitivity in patients with impairment in star cancellation, whereas the subgroups with and without impairment in the star cancellation task of our sample did not differ in perceptual sensitivity pre training. Currently, we have no explanation for the difference, yet. Concerning the subgroups with and without impaired gesture imitation, the present study revealed no significant pre training differences in perceptual sensitivity in the Aperture Task which again is in accordance with Randerath and colleagues [1]. Overall, most results were replicated for assessing AJ performance in the Aperture Task.

1. Randerath J, Finkel L, Shigaki C, Burris J, Nanda A, Hwang P, et al. Does it fit? – Impaired affordance perception after stroke. Neuropsychologia. 2018;108:92-102. doi: 10.1016/j.neuropsychologia.2017.11.031.
